# Allogeneic CD4 T Cells Sustain Effective BK Polyomavirus-Specific CD8 T Cell Response in Kidney Transplant Recipients

**DOI:** 10.1016/j.ekir.2024.04.070

**Published:** 2024-05-07

**Authors:** Manon Dekeyser, Marie-Ghislaine de Goër de Herve, Houria Hendel-Chavez, Romain Lhotte, Ivan Scriabine, Karen Bargiel, Emmanuelle Boutin, Florence Herr, Jean-Luc Taupin, Yassine Taoufik, Antoine Durrbach

**Affiliations:** 1 INSERM 1186, Gustave Roussy Institute, Villejuif, France; 2 Paris-Saclay University, Paris, France; 3 Department of Nephrology, Center Hospitalier Régional Universitaire d'Orléans, Orléans, France; 4 Laboratory of Immunology and Histocompatibility, Saint Louis Hospital, Assistance Publique-Hôpitaux de Paris, INSERM U976 (Team 3), Paris, France; 5 Unit of Clinical Research, Henri Mondor Hospital, Assistance Publique-Hôpitaux de Paris, Creteil, France; 6 Paris Est Creteil University, INSERM, IMRB, CEpiA Team, Creteil, France; 7 Department of Nephrology and Transplantation, Henri Mondor Hospital, Assistance Publique-Hôpitaux de Paris, Creteil, France

**Keywords:** BK polyomavirus reactivation, BK polyomavirus-associated nephropathy, BK polyomavirus-specific T cell, donor-recipient HLA divergence, kidney transplantation

## Abstract

**Introduction:**

BK polyomavirus-associated nephropathy (BKPyVAN) is a significant complication in kidney transplant recipients (KTRs), associated with a higher level of plasmatic BK polyomavirus (BKPyV) replication and leading to poor graft survival.

**Methods:**

We prospectively followed-up with 100 KTRs with various degrees of BKPyV reactivation (no BKPyV reactivation, BKPyV-DNAuria, BKPyV-DNAemia, and biopsy-proven BKPyVAN [bp-BKPyVAN], 25 patients per group) and evaluated BKPyV-specific T cell functionality and phenotype.

**Results:**

We demonstrate that bp-BKPyVAN is associated with a loss of BKPyV-specific T cell proliferation, cytokine secretion, and cytotoxic capacities. This severe functional impairment is associated with an overexpression of lymphocyte inhibitory receptors (programmed cell death 1 [PD1], cytotoxic T lymphocyte-associated protein 4, T cell immunoreceptor with Ig and ITIM domains, and T cell immunoglobulin and mucin domain-containing-3), highlighting an exhausted-like phenotype of BKPyV-specific CD4 and CD8 T cells in bp-BKPyVAN. This T cell dysfunction is associated with low class II donor-recipient human leukocyte antigen (HLA) divergence. In contrast, in the context of higher class II donor-recipient HLA (D/R-HLA) divergence, allogeneic CD4 T cells can provide help that sustains BKPyV-specific CD8 T cell responses. *In vitro*, allogeneic HLA-mismatched CD4 T cells rescue BKPyV-specific CD8 T cell responses.

**Conclusion:**

Our findings suggest that in KTRs, allogeneic CD4 T cells can help to maintain an effective BKPyV-specific CD8 T cell response that better controls BKPyV replication in the kidney allograft and may protect against BKPyVAN.

## Graphical abstract

**Figure:**
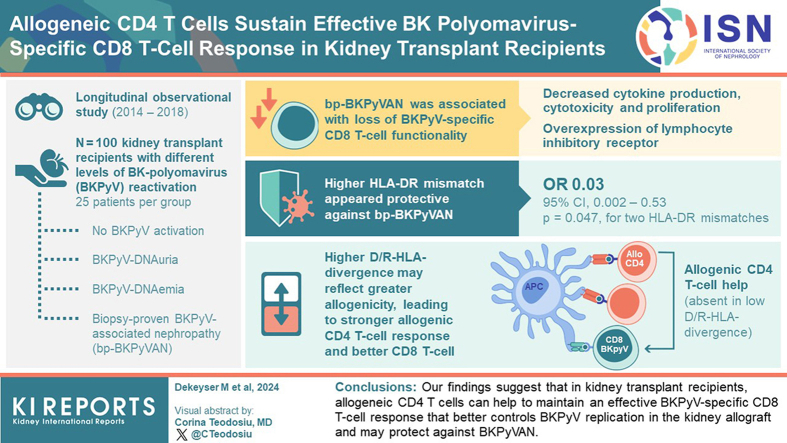

BKPyV represents a major cause of opportunistic infections in KTRs.^1^^,^^2^ BKPyV reactivation occurs in 30% to 40% of KTRs. It may lead to BKPyVAN, which affects almost 10% of KTRs and may result in premature kidney allograft failure.^3^, ^4^, ^5^ BKPyV replication progresses gradually in KTRs. The initial event is BKPyV replication in tubular epithelial cells, with virus excreted in urine (BKPyV-DNAuria). Without viral control, progression to viremia may be observed (BKPyV-DNAemia), which is associated in some cases with BKPyVAN. Whereas low-level BKPyV-DNAuria has been described in healthy individuals,^6^ high-level BKPyV-DNAuria has been identified as a precursor to BKPyV-DNAemia. BKPyVAN is characterized by viral replication in kidney epithelial cells with intranuclear viral inclusion, cell dysmorphia, eventually leading to cell lysis and the desquamation of tubular epithelial cells in urine. The lesions are also associated with interstitial fibrosis, tubular atrophy, and mononuclear cell infiltration. Moreover, patients with sustained high-level BKPyV-DNAemia are considered to have kidney allograft involvement, because focal BKPyV replication could be missed by needle biopsy. Thus, in recent international guidelines, probable, presumptive, and bp-BKPyVAN have been proposed for the clinical staging of BKPyV replication to initiate therapeutical modifications required to reduce BKPyV-DNAemia, even without renal dysfunction.^1^^,^^7^

Currently, there is no specific antiviral treatment for BKPyV infection. The virus control by CD8 T cells leads to the clearance of infected cells, rapidly replaced by newly differentiated epithelial cells.^1^^,^^2^ Therefore, the primary therapeutic option is to decrease the level of immunosuppression in order to restore BKPyV-specific immune responses. However, this approach entails a risk of allogeneic graft rejection.^8^ Therefore, managing BKPyV replication and BKPyV-specific immune restoration is a significant challenge in kidney transplantation. In addition, the BKPyV-specific T cell functions in KTRs developing different stages of BKPyV replications have been merely studied. A better characterization of their status must be precise, particularly in patients with BKPyVAN.

BKPyV is a widespread double-stranded DNA polyomavirus, with seroprevalence rates of up to 80% in adults worldwide.^6^^,^^9^ Primary infection frequently occurs during childhood, after which BKPyV establishes viral latency in the reno-urinary tract, where the BKPyV-specific T cell response controls the virus.^9^ The early-gene region of the viral genome encodes proteins involved in viral replication, such as the “large-tumor antigen.” The late-gene region encodes capsid proteins, such as viral protein-1.^10^ Large-tumor antigen and viral protein-1 are immunodominant proteins widely recognized by BKPyV-specific T cells, which contribute to the control of BKPyV.^11^ Therefore, monitoring the BKPyV-specific T cell response and BKPyV load may help to assess the risk of BKPyV reactivation and development of BKPyVAN.

We prospectively studied BKPyV-specific T cell responses, their exhausted-like phenotype, and the impact of HLA matching in a cohort of KTRs with various levels of BKPyV reactivation (no BKPyV reactivation, BKPyV-DNAuria, BKPyV-DNAemia, bp-BKPyVAN, 25 patients per group).

## Methods

### Study Design

We performed a longitudinal observational study in the Kidney Transplant Department in AP-HP University Hospitals. We prospectively included 100 KTRs in 4 groups (25 patients/group) based on the level of BKPyV reactivation and followed-up with them between April 2014 and April 2018 as follows:

1. Patients with BKPyV-DNAuria (urine BKPyV load > 200 copies/ml and plasma BKPyV load < 200 copies/ml in the last 12 months).
2. Patients with BKPyV-DNAemia (stable or increasing plasma BKPyV load > 200 copies/ml in the last 6 months, without BKPyVAN diagnosis on kidney biopsy).
3. Patients with bp-BKPyVAN with a plasma BKPyV load > 200 copies/ml.
4. Patients without BKPyV reactivation (plasma and urine BKPyV loads < 200 copies/ml in the last 12 months) as KTR group control.

The patients without BKPyV reactivation and with BKPyV-DNAuria were selected randomly from our database of 470 patients. The study was approved by the local Health Service Research Ethics Committee (Health Service Research Ethics Committee Ile de France VII ref. PP14-046).

### Clinical, Biological, and Histological Data

Demographic, clinical, and histological data were recorded for all patients (Table 1). BKPyV-DNA quantification was performed by BKPyV-specific real-time polymerase chain reaction following international guidelines (Supplementary Methods).^1^^,^^2^

**Table 1:** Demographics of the study population, with baseline characteristics, comparison of immunosuppressive treatments and outcome after kidney transplantation

Demographic comparison	Without BKPyV	BKPyV-DNAuria	BKPyV-DNAemia	Bp-BKPyVAN	*P*-value^a^
*n*	25	25	25	25	
Age of patients at inclusion, yr	49 (39–63)	59 (47–67)	62 (49–70)	60 (54–64)	0.129
Male, *n* (%)	16 (64)	18 (72)	18 (72)	17 (68)	0.916
Dialysis, *n* (%)	20 (80)	23 (92)	23 (92)	23 (92)	0.431
Time on dialysis, mo	32 (16.8–46)	28 (11–43)	40 (20.8–61.8)	47 (28–74)	0.137
Hemodialysis, *n* (%)	18 (90)	19 (82.6)	21 (91.3)	22 (95.7)	0.526
Peritoneal dialysis, *n* (%)	2 (10)	4 (17.4)	2 (8.7)	1 (4.3)	
Cause of end-stage renal disease					
Glomerulonephritis, *n* (%)	6 (24)	10 (40)	8 (32)	6 (24)	0.611
Hypertensive or diabetic nephrosclerosis, *n* (%)	5 (20)	7 (28)	10 (40)	9 (36)	
Polycystic kidneys, *n* (%)	6 (24)	3 (12)	3 (12)	2 (8)	
Tubular or interstitial diseases, *n* (%)	2 (8)	1 (4)	1 (4)	4 (16)	
Unknown and other causes, *n* (%)	6 (24)	4 (16)	3 (12)	4 (16)	
Transplantation characteristics					
Donation after brain death, *n* (%)	16 (64)	21 (84)	17 (68)	20 (80)	0.319
Donation after cardiac death, *n* (%)	4 (16)	0 (0)	5 (20)	2 (8)	
Living donation, *n* (%)	5 (20)	4 (16)	3 (12)	3 (12)	
Highly sensitized patients^b^, *n* (%)	7 (28)	6 (24)	6 (24)	5 (20)	0.932
First transplant, *n* (%)	19 (76)	22 (88)	24 (96)	19 (76)	0.147
Preexisting DSA, *n* (%)	10 (40)	8 (32)	10 (40)	10 (40)	0.917
*De novo* DSA status, *n* (%)	9 (36)	7 (28)	7 (28)	5 (20)	0.662
CMV R+ status, *n* (%)	17 (68)	22 (88)	22 (88)	19 (76)	0.212
CMV D+/R- status, *n* (%)	4 (16)	1 (4)	2 (8)	3 (12)	0.528
Total (HLA-A, -B, -DR, -DQ) mismatch number	5 (3–7)	4 (3–6)	6 (5–7)	4 (3–6)	*0.030*
HLA-A mismatch number	1 (1–2)	1 (1–1.5)	1 (1–2)	1 (0–2)	0.788
HLA-B mismatch number	1 (1–2)	2 (1–2)	2 (2–2)	2 (1–2)	0.040
HLA-DR mismatch number	1 (1–2)	1 (0–1)	2 (1–2)	1 (0–1.5)	0.0036
HLA-DQ mismatch number	1 (0–2)	1 (0–2)	1 (1–2)	1 (0–1)	0.459
BKPyV reactivation					
Plasma BKPyV viral load (copies/ml) at the inclusion		0	2.2 × 10^3^ (8.9 × 10^2^ to 1.2 × 10^4^)	3.5 × 10^5^ (5.0 × 10^4^ to 7.8 × 10^5^)	<0.0001
Time from kidney transplantation to plasma BKPyV reactivation, mo			5.6 (3.2–9.1)	3.4 (2.9–13.4)	0.859
Urinary BKPyV viral load (copies/ml) at the inclusion		2.4 × 10^4^ (3.9 × 10^3^ to 2.9 × 10^5^)	8.5 x 10^7^ (2.2 × 10^6^ to 2.9 × 10^8^)	1.0 × 10^9^ (1.9 × 10^8^ to 1.6 × 10^9^)	<0.0001
Time from kidney transplantation to urinary BKPyV reactivation, mo		14.8 (2.6–51)	3.9 (1.7–11)	3.6 (1.7–13.4)	0.047
Biopsy-proven BKPyVAN diagnosis, mo				11 (5–24)	
Induction and maintenance treatments					
Thymoglobulin, *n* (%)		6 (24)	13 (52)	15 (60)	0.027
Tacrolimus, *n* (%)		19 (76)	16 (64)	21 (84)	0.516
Levels of tacrolimus, ng/ml		9.1 (8.7–9.7)	9.1 (7.7–11.1)	8.6 (6.7–10.2)	0.670
Mycophenolate mofetil exposure, AUC h.mg/l		31.5 (24.4–44.6)	44 (41–53)	58 (51–74.5)	0.0005
Curative treatment for rejection					
Intravenous steroid boluses, *n* (%)		5 (20)	9 (36)	10 (40)	0.130
Thymoglobulin, *n* (%)		1 (4)	4 (16)	1 (4)	>0.9999
Plasma exchanges, *n* (%)		4 (16)	8 (32)	6 (24)	0.508
Rituximab, *n* (%)		4 (16)	8 (32)	4 (16)	>0.9999
Bortezomib, *n* (%)		5 (20)	3 (12)	4 (16)	0.743
Outcome after transplantation					
Delayed graft function, *n* (%)	11 (44)	6 (24)	7 (28)	8 (32)	0.462
eGFR at month one after transplantation, ml/min per 1.73 m^2^	40 (32.3–68.8)	51 (31–81)	45.5 (31.3–63.5)	41 (26.8–56.8)	0.740
eGFR at biopsy-proven BKPyVAN diagnosis or 12 mo after transplantation, ml/min per 1.73 m^2^	51 (42.5–69.5)	43 (35–69.5)	48 (38–56)	29 (21–42.5)	<0.0001
eGFR at the end of the follow-up, ml/min per 1.73 m^2^	49 (37–67.5)	47 (31.5–58.5)	45 (28.5–60)	13 (9.5–29)	<0.0001
Decrease in eGFR at the end of follow-up, ml/min per 1.73 m^2^	0 (0–7)	3 (0–6.5)	4 (0–13)	12 (3.5–17)	0.0079
CMV reactivation, *n* (%)	10 (40)	8 (32)	7 (28)	9 (36)	0.828
EBV reactivation, *n* (%)	13 (52)	18 (72)	16 (64)	15 (60)	0.531
Graft rejection, *n* (%)	6 (24)	5 (20)	9 (36)	4 (16)	0.381
Graft loss, *n* (%)	0 (0)	0 (0)	1 (4)	9 (36)	<0.0001
Patient death after transplantation, *n* (%)	1 (11.1)	3 (33.3)	2 (22.2)	3 (33.3)	0.720
Time from transplantation to inclusion, mo	26 (16–34)	27 (9–79)	9 (7–18)	8 (4–28)	0.0014
CD4 T cell count, absolute number/μl	247 (137–514)	342 (260–495)	253 (156–443)	210 (76–376)	0.472
CD8 T cell count, absolute number/μl	210 (161–271)	311 (162–448)	172 (92–278)	159 (122–353)	0.197

AUC, area under the curve; BKPyV, BK polyomavirus; BKPyV-DNAemia, kidney transplant recipients with plasma BKPyV reactivation; BKPyV-DNAuria, kidney transplant recipients with urinary BKPyV reactivation; Bp-BKPyVAN, kidney transplant recipients with biopsy-proven BKPyV-associated nephropathy; CMV D+/R -status, recipient seronegative for CMV transplanted with a graft from a CMV-seropositive donor; CMV R+ status, recipient seropositive for CMV; CMV, cytomegalovirus; DSA, donor-specific alloantibodies; EBV, Epstein Barr virus; eGFR, estimated glomerular filtration rate (Modification of Diet in Renal Disease-MDRD4 -formula); HLA, human leukocyte antigen; *n,* number of patients; NA, not applicable; Without BKPyV, kidney transplant recipients without plasma or urinary BKPyV reactivation.

Continuous data are expressed as medians and interquartile ranges.

The immunosuppressive treatments were compared between groups at biopsy-proven BKPyVAN diagnosis or 12 months after transplantation.

a *P*-values indicate the significance of differences between groups in Kruskal-Wallis or χ^2^ tests.

b Defined as >85% panel reactive antibodies.

Glomerular filtration rate was estimated with the Modification of Diet in Renal Disease-4 formula at 1 month, at bp-BKPyVAN diagnosis or 12 months posttransplantation, and at the end of follow-up. We recorded levels of tacrolimus, everolimus, and mycophenolate mofetil exposure (area under the curve in h.mg/l) before and after BKPyVAN diagnosis for bp-BKPyVAN group, or 12 months posttransplantation for other groups.

Kidney biopsy was performed at 3 and 12 months posttransplantation or in cases of acute kidney injury. Bp-BKPyVAN was diagnosed histologically following international guidelines and confirmed by immunohistochemistry (large-SV40 tumor-antigen).^12^^,^^13^

### HLA Matching and D/R-HLA Divergence

The degree of HLA matching was determined for each donor-recipient combination (Supplementary Methods). Antigenic mismatches within each locus were counted as 0, 1, or 2.

The distance between 2 pairs of alleles was calculated as described in the Supplementary Methods. We assessed D/R-HLA divergence by calculating the Grantham score,^14^ usually used to quantify the divergence between 2 homologous alleles from an individual’s single locus.^15^, ^16^, ^17^ The Grantham score^14^ evaluates the quantitative “pairwise” distance between an individual’s HLA alleles, also called HLA-evolutionary divergence. This quantitative “pairwise” distance allows to consider the physiochemical properties of amino acids; thus, the functional similarity. It reflects the breadth of the immunopeptidome, defined as the set of peptides presented by major histocompatibility complex molecules on the surface of antigen-presenting cells to enable T cell activation.

### BKPyV-Specific T cell Functionality and Phenotype

We assessed T cell functionality and phenotype as previously described^18^^,^^19^ on flow cytometer (LSR-Fortessa, BD-Biosciences) using FlowJo.v10 software (Supplementary Methods). All patient’s T cell assessments were evaluated at inclusion time. Cryopreservation conditions for blood sampling were similar for all patients.

Cryopreserved peripheral blood mononuclear cells (PBMCs) were thawed and cultured in a 96-well plate. For BKPyV activation, PBMCs were incubated with 2 different BKPyV-specific antigens (PepTivator BKV LT-Ag or viral protein-1, 1 μg/ml/peptide, Miltenyi) activating both CD4 and CD8 T cells. To confirm the specificity of the BKPyV-specific antigenic activation, we isolated BKPyV-specific CD8 T cells by using BKPyV-pentamers from a subgroup of HLA-A2 or HLA-B7 KTRs (ProImmune, Supplementary Methods).

As global antiviral immune control, we used CEF peptides (a peptide pool from cytomegalovirus, Epstein-Barr, and influenza viruses, 1 μg/ml/peptide, AXXORA). All activations were performed relative to a negative control (unstimulated cells) and a positive control (staphylococcal-enterotoxin-B, 0.2 μg/ml, Sigma-Aldrich).

The T cell proliferation was assessed by carboxifluorescein succinimidyl ester (CFSE) dilution by flow cytometry (Supplementary Figure S1A).

The T cell cytokine production was assessed by interferon-γ (IFNγ) and tumor necrosis factor-α (TNFα) staining. PBMCs (4 × 10^6^/condition) were incubated overnight in the presence of brefeldin-A (1 μl/ml, BD). PBMCs were stained using antihuman CD3, CD4, and CD8 antibodies, fixed, and permeabilized using the Cytofix/Cytoperm reagent (BD). PBMCs were stained using anti-human IFNγ and TNFα antibodies (Supplementary Figure S1A).

The T cell cytotoxicity was assessed by the mortality rate of target cells. Total CD8 T cells were incubated overnight with autologous target cells (CD8-depleted PBMCs) (ratio, 3:1).^18^^,^^20^ Target cells were distinguished from CD8 T cells by CFSE staining (0.5 μM). The mortality of target cells was evaluated using 7-aminoactinomycin-D staining (0.5 μM) (Supplementary Figure S1A).

The T cell phenotype was assessed using antihuman CD3, CD4, CD8, and T cell inhibitory receptors (PD1, cytotoxic T lymphocyte-associated protein 4, T cell immunoreceptor with Ig and ITIM domains; T cell immunoglobulin and mucin domain-containing-3) antibodies (Supplementary Figure S2).

### BKPyV-Specific T cell Proliferation in the Presence of Allogeneic CD4 T Cell Help

We assessed lymphocyte proliferation in response to BKPyV-peptides in the presence and absence of allogeneic CD4 T cells. PBMCs from 13 KTRs with bp-BKPyVAN were stained with a fluorescent dye, depleted of autologous CD4 T cells (CD4-magnetic depletion), and incubated for 5 days with BKPyV-specific peptide pools in the presence of autologous or allogeneic third-party CD4 T cells (ratio, 1 CD4 / 5 non-CD4 T cells). We distinguished PBMCs from allogeneic third-party cells by using fluorescent dyes (CFSE or Violet Proliferation Dye 450, 0.5 μm). Unstimulated CD4-depleted PBMCs incubated in the presence of autologous CD4 T cells, or allogeneic third-party CD4 T cells were used as controls. After 5 days, stimulated cells were stained for CD3, CD4, CD8 antibodies, and their proliferation capacity was evaluated by fluorescent dye dilution (CFSE or Violet Proliferation Dye 450).

### Statistical Analysis

For categorical data, percentages were used and compared using chi-square tests. Kaplan-Meier survival curves were compared in log-rank tests. For continuous data, medians and interquartile ranges were used. To normalize the data distribution, the frequencies of BKPyV-specific T cells were subjected to natural logarithm or square root transformation. As appropriate, comparisons between 2 groups were performed with nonparametric Mann-Whitney or Wilcoxon matched-pairs tests. More than 2 groups were compared using nonparametric Kruskal-Wallis tests, followed by Dunn’s test for multiple comparisons. Correlations were evaluated with the nonparametric Spearman tests. Odds ratios with 95% confidence intervals were compared by univariate multinomial logistic regression with correction for false discovery rate. Multivariate analysis was performed with Wald tests (multivariate multinomial logistic regression). Differences were considered significant if *P* < 0.05. Statistical analyses were performed with STATA.v15 software (StataCorp, College Station, TX) and graphs were done with GraphPad.Prism.v9 software (GraphPad Prism, La Jolla, CA).

## Results

We examined 100 KTRs with various degrees of BKPyV reactivation (no BKPyV reactivation, BKPyV-DNAuria, BKPyV-DNAemia, and bp-BKPyVAN, 4 groups of 25 patients each). Patients were followed-up with prospectively for a median of 36 (26–38) months.

### High Levels of Plasma BKPyV Loads and Poor Graft Survival in Patients With bp-BKPyVAN

We analyzed clinical, histological, and biological parameters in KTR patients before and at the time of transplantation and for 36 months following study inclusion (Table 1). Before and at the time of transplantation, we found no significant differences between the KTR groups. There were no significant differences in graft function (based on estimated glomerular filtration rate) during the first month after transplantation, suggesting a similar quality of grafts and that the rate of graft allogeneic rejection was similar (Table 1).

**Figure 1:**
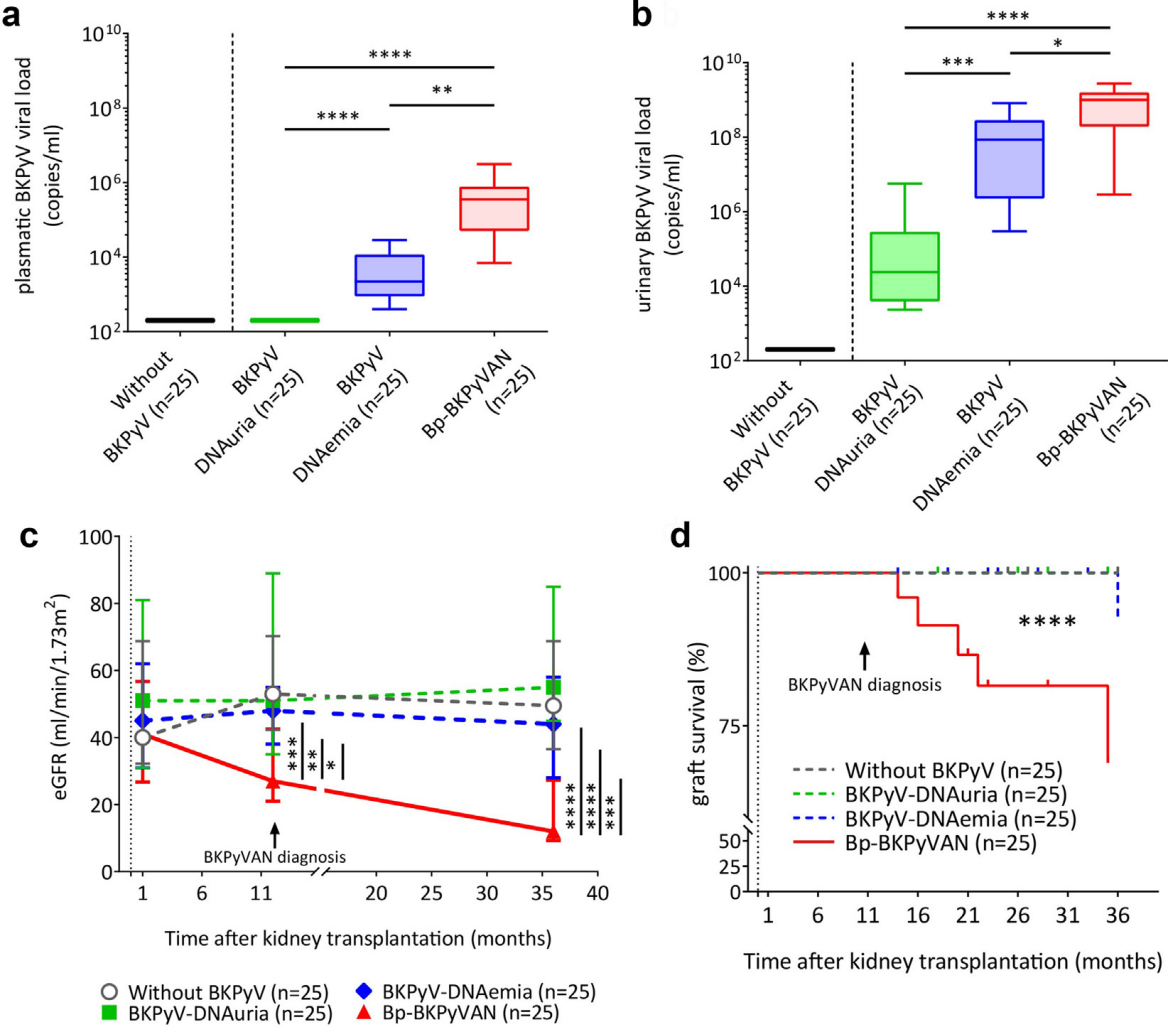
High levels of plasma BKPyV loads and poor graft survival in KTRs with biopsy-proven BKPyVAN. We examined clinical and biological features in 4 groups of KTR according to the stage of BKPyV reactivation (KTRs without BKPyV reactivation, KTRs with BKPyV-DNAuria, KTRs with BKPyV-DNAemia and KTRs with bp-BKPyVAN; 25 KTRs by group). (a) and (b) show plasma and urinary BKPyV loads. **(**c) shows graft function in KTR groups. Graft function was evaluated by eGFR (Modification of Diet in Renal Disease-4 formula) at 3 time points: 1-month, BKPyVAN diagnosis or 12 months posttransplantation, and at the end of the follow-up. (d) shows graft survival. Statistical analysis was performed by using Kruskal-Wallis tests followed by Dunn’s multiple comparison tests between the BKPyV-DNuria, BKPyV-DNAemia, or bp-BKPyVAN KTRs (a and b) nonparametric repeated-measures 2-way analysis of variance (c), and Kaplan-Meier survival curves with log-rank (d) tests. Boxes represent the median and 25th and 75th percentiles; whiskers represent the 10th and 90th percentiles. eGFRs are represented as the median and interquartile range. ∗*P* < 0.05, ∗∗*P* < 0.005, ∗∗∗*P* < 0.0005, ∗∗∗∗*P* < 0.0001. BKPyV, BK polyomavirus; BKPyVAN, BK polyomavirus-associated nephropathy; bp-BKPyVAN, biopsy-proven BKPyVAN; eGFR, estimated glomerular filtration rate; KTR, kidney transplant recipient.

At study inclusion, the bp-BKPyVAN group had higher plasma BKPyV viral loads than the BKPyV-DNAemia group (Figure 1a, *P* = 0.0019). Similarly, bp-BKPyVAN-KTRs had higher urinary BKPyV viral loads than those with BKPy-DNAuria or BKPy-DNAemia (Figure 1b, *P* < 0.0001 and *P* = 0.022, respectively).

The median time from transplantation to bp-BKPyVAN diagnosis was 11 (5–24) months. Despite having a similar renal function 1 month after transplantation, bp-BKPyVAN KTRs had poorer renal function than those of the other groups at month 12 (*P* < 0.0001), highlighting the impact of the occurrence of bp-BKPyVAN on the graft function (Figure 1c, Table 1). In contrast, no significant decrease in estimated glomerular filtration rate was found in patients from the other groups between the inclusion and the end of follow-up (Figure 1c, Table 1). Immunosuppressive treatment was adapted for patients, with a decrease in tacrolimus dose, a reduction or withdrawal of antimetabolite exposure, and in some cases, a switch to mammalian target of rapamycin inhibitor (Supplementary Table S1). Bp-BKPyVAN KTRs had poorer graft survival, with a graft loss rate of 36% versus <5% in other KTR groups (*P* < 0.0001) (Figure 1d, Table 1).

### Loss of BKPyV-Specific CD8 Functionality in Patients With bp-BKPyVAN

We examined potential risk factors associated with bp-BKPyVAN. More patients received induction with thymoglobulin in the bp-BKPyVAN group than in other groups (*P* = 0.027) and had higher mycophenolate mofetil exposure (area under the curve) (*P* = 0.0005). However, at the time of BKPyVAN diagnosis, CD4 and CD8 T cell counts were similar between KTR groups (Table 1).

The BKPyV-DNAemia group had a higher number of HLA-DR mismatches than in other KTR groups (*P* = 0.0036) (Table 1). Conversely, bp-BKPyVAN-KTRs had a lower number of HLA-DR mismatches than patients from the viremia group (*P* = 0.039) but not as compared to patients with only the BKPyV-DNAuria group. Mismatches at other loci, HLA-A, HLA-B, and HLA-DQ, did not differ significantly between KTR groups (Table 1).

We next examined the BKPyV-specific T cell responses after activation with BKPyV or CEF peptides. In most KTRs without BKPyV reactivation, CD8 T cells were able to proliferate, produce cytokines, and develop CD8 cytotoxicity in the presence of BKPyV or CEF-specific peptides, showing a high functionality of BKPyV and CEF-specific CD8 T cell responses (Supplementary Figure S1B and Supplementary Table S2).

**Figure 2:**
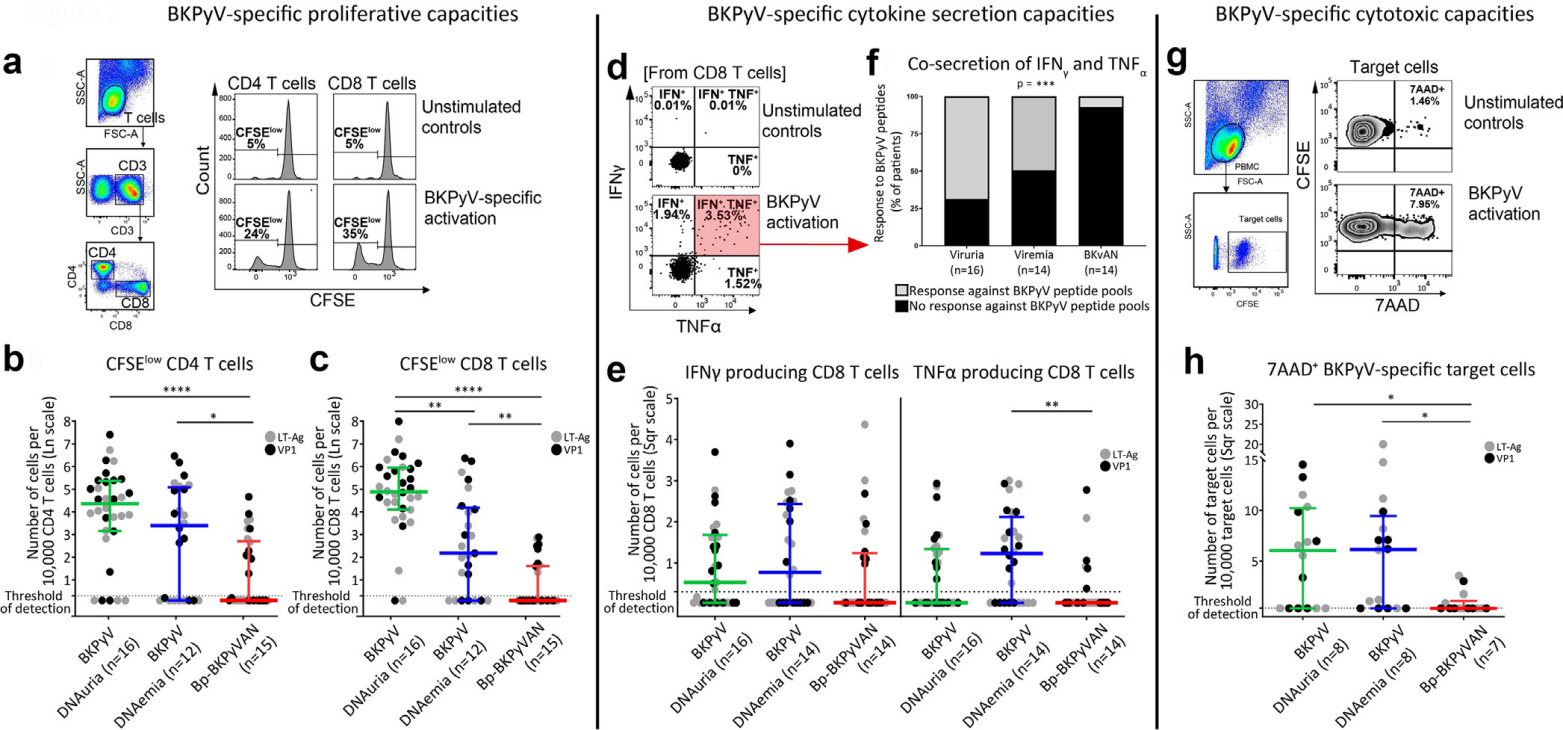
Loss of BKPyV-specific CD8 functionality in KTRs with biopsy-proven BKPyVAN. We examined BKPyV-specific T cell responses in KTR groups according to BKPyV reactivation levels. (a and b) show proliferative capacities of BKPyV-specific CD4 T cells and BKPyV-specific CD8 T cells, respectively. (c) shows representative plots of CD4 and CD8 T cell gating and CFSE dilution following activation with BKPyV peptide pools for 5 days. (d) shows IFNγ and TNFα production capacities of BKPyV-specific CD8 T cells. (e) shows representative plots of coproduction of IFNγ and TNFα in CD8 T cells after overnight activation with BKPyV peptide pools. (f) shows the proportion of KTRs able to perform coproduction of IFNγ and TNFα. (g) shows cytotoxic capacities of BKPyV-specific CD8 T cells. (h) shows representative plots of 7AAD expression on autologous target cells (CD8-depleted PBMCs loaded with BKPyV peptides) in contact with CD8 T cells activated overnight with BKPyV peptide pools. BKPyV-specific CD4 or CD8 T cells were expressed as the number of cells per 10,000 CD4 or CD8 T cells and then subjected to natural logarithm or square root transformation to normalize the data distributions. *n* represents the number of patients. Two BKPyV-specific responses were analyzed for each patient, after activation with 2 BKPyV-specific peptide pools (LT-Ag and VP1-peptides). Statistical analysis was performed (a, b, d, and g) using Kruskal-Wallis tests followed by Dunn’s multiple comparison tests or (f) by χ^2^ for trend test. Scatter dot plots are shown, with the median and interquartile range. ∗*P* < 0.05, ∗∗*P* < 0.005, ∗∗∗*P* < 0.0005, ∗∗∗∗*P* < 0.0001. 7AAD, 7-Aminoactinomycin D; BKPyV, BK polyomavirus; BKPyVAN, BK polyomavirus-associated nephropathy; IFNγ, interferon-γ; KTR, kidney transplant recipient; LT-Ag, large tumor antigen; PBMC, peripheral blood mononuclear cell; TNFα, tumor necrosis factor-α; VP1, viral protein-1.

BKPyV-specific CD4 T cells proliferated better following antigen activation in the BKPyV-DNAemia group as compared to bp-BKPyVAN KTRs, whereas no difference was found as regards cytokine production (Figure 2a and b, Table 2, and Supplementary Figure S3).

**Table 2:** Comparison of BKPyV-specific T cell functionalities between KTR groups defined on the basis of BKPyV reactivation-BKPyV-specific T cell functionality

BKPyV-specific T cell functionality	BKPyV-DNAuria	BKPyV-DNAemia	Bp-BKPyVAN	*P*-value^a^
Proliferation capacities (*n*)	16	13	15	
CFSE low BKPyV (LT-Ag and VP1) CD4 T cells, cell nb	4.36 (3.15–5.37)	3.39 (0.10–5.09)	0.10 (0.10–2.70)	<0.0001
Patients without BKPyV-specific peptide response, *n* (%)	2 (12.5)	2 (16.7)	6 (40.0)	0.07
Patients with BKPyV-specific peptide response, *n* (%)	14 (87.5)	10 (83.3)	9 (60.0)	
Correlation between LT-Ag CD4 T cells and BKPyV viral load		0.08 (−0.32 to 0.46)		0.69
Correlation between VP1 CD4 T cells and BKPyV viral load		−0.59 (−0.80 to −0.26)		0.001
CFSE low BKPyV (LT-Ag and VP1) CD8 T cells, cell nb	4.88 (4.10–5.95)	2.18 (0.10–4.18)	0.10 (0.10–1.61)	<0.0001
Patients without BKPyV-specific peptide response, *n* (%)	0 (0)	2 (16.7)	7 (87.5)	<0.0001
Patients with BKPyV-specific peptide response, *n* (%)	16 (100)	10 (83.3)	1 (12.5)	
Correlation between LT-Ag CD8 T cells and BKPyV viral load		−0.35 (−0.65; 0.04)		0.07
Correlation between VP1 CD8 T cells and BKPyV viral load		−0.44 (−0.71; −0.06)		0.021
Cytokine secretion capacities (*n*)	16	14	14	
IFN^+^ BKPyV (LT-Ag and VP1) CD4 T cells, cell nb	0.03 (0.03–1.95)	1.30 (0.03–2.01)	0.37 (0.03–2.45)	0.720
Patients without BKPyV-specific peptide response, *n* (%)	6 (37.5)	5 (35.7)	5 (38.5)	0.96
Patients with BKPyV-specific peptide response, *n* (%)	10 (62.5)	9 (64.3)	8 (61.5)	
Correlation between LT-Ag CD4 T cells and BKPyV viral load		−0.07 (−0.45 to 0.34)		0.751
Correlation between VP1 CD4 T cells and BKPyV viral load		0.14 (−0.26 to 0.49)		0.494
TNF^+^ BKPyV (LT-Ag and VP1) CD4 T cells, cell nb	0.53 (0.03–2.29)	1.50 (0.03–1.94)	0.03 (0.03–1.22)	0.115
Patients without BKPyV-specific peptide response, *n* (%)	5 (31.3)	5 (35.7)	6 (42.9)	0.803
Patients with BKPyV-specific peptide response, *n* (%)	11 (68.7)	9 (64.3)	8 (57.1)	
Correlation between LT-Ag CD4 T cells and BKPyV viral load		−0.16 (−0.52 to 0.25)		0.433
Correlation between VP1 CD4 T cells and BKPyV viral load		−0.23 (−0.57 to 0.16)		0.232
TNF^+^ & IFN^+^ BKPyV (LT-Ag and VP1) CD4 T cells, cell nb	0.86 (0.03–1.29)	0.60 (0.03–1.27)	0.03 (0.03–0.81)	0.023
Patients without BKPyV-specific peptide response, *n* (%)	2 (12.5)	3 (21.4)	5 (35.7)	0.132
Patients with BKPyV-specific peptide response, *n* (%)	14 (87.5)	11 (78.6)	9 (64.3)	
Correlation between LT-Ag CD4 T cells and BKPyV viral load		−0.20 (−0.54 to 0.20)		0.302
Correlation between VP1 CD4 T cells and BKPyV viral load		−0.38 (−0.67 to 0.00005)		0.044
IFN^+^ BKPyV (LT-Ag and VP1) CD8 T cells, cell nb	0.52 (0.03–1.63)	0.77 (0.03–2.43)	0.03 (0.03–1.24)	0.306
Patients without BKPyV-specific peptide response, *n* (%)	4 (25)	5 (35.7)	6 (42.9)	0.301
Patients with BKPyV-specific peptide response, *n* (%)	12 (75)	9 (64.3)	8 (57.1)	
Correlation between LT-Ag CD8 T cells and BKPyV viral load		−0.13 (−0.49 to 0.27)		0.517
Correlation between VP1 CD8 T cells and BKPyV viral load		−0.02 (−0.40 to 0.37)		0.941
TNF^+^ BKPyV (LT-Ag and VP1) CD8 T cells, cell nb	0.03 (0.03–1.33)	1.23 (0.03–2.11)	0.03 (0.03–0.03)	0.0018
Patients without BKPyV-specific peptide response, *n* (%)	7 (43.8)	3 (21.4)	9 (64.3)	0.073
Patients with BKPyV-specific peptide response, *n* (%)	9 (56.2)	11 (78.6)	5 (35.7)	
Correlation between LT-Ag CD8 T cells and BKPyV viral load		−0.71 (−0.86 to −0.44)		<0.0001
Correlation between VP1 CD8 T cells and BKPyV viral load		−0.53 (−0.76 to −0.19)		0.0035
TNF^+^ & IFN^+^ BKPyV (LT-Ag and VP1) CD8 T cells, cell nb	0.03 (0.03–0.54)	0.03 (0.03–0.59)	0.03 (0.03–0.03)	0.0011
Patients without BKPyV-specific peptide response, *n* (%)	5 (31.3)	7 (50)	13 (92.9)	0.0007
Patients with BKPyV-specific peptide response, *n* (%)	11 (68.7)	7 (50)	1 (7.1)	
Correlation between LT-Ag CD8 T cells and BKPyV viral load		−0.39 (−0.67 to −0.006)		0.041
Correlation between VP1 CD8 T cells and BKPyV viral load		−0.24 (−0.57 to 0.16)		0.23
Cytotoxic capacities (*n*)	8	8	7	
7AAD^+^ BKPyV (LT-Ag and VP1) target cells (cell nb)	6.04 (0.32–10.22)	6.13 (0.32–9.45)	0.32 (0.32–1.07)	0.012
Patients without BKPyV-specific peptide response, *n* (%)	2 (25)	1 (12.5)	4 (57.1)	0.195
Patients with BKPyV-specific peptide response, *n* (%)	6 (75)	7 (87.5)	3 (42.9)	

7AAD, 7-Aminoactinomycin D; BKPyV, BK polyomavirus; BKPyV-DNAemia, kidney transplant recipients with plasma BKPyV reactivation; BKPyV-DNAuria, kidney transplant recipients with urinary BKPyV reactivation; Bp-BKPyVAN, kidney transplant recipients with biopsy-proven BKPyV-associated nephropathy; Epstein Barr virus and influenza virus peptides; cell nb, normalized BKv-specific T-cell to 10,000 T cell corresponding CD4 or CD8 T cells; IFN, interferon; LT-Ag, large tumor antigen; *n*, number of patients; NA, not applicable; TNF, tumor necrosis factor; VP1, viral protein-1.

Continuous data are expressed as medians and interquartile ranges.

Correlations were evaluated with the nonparametric Spearman’s rank correlation test.

For each patient, 2 BKPyV-specific responses were analyzed after stimulation with LT-Ag and VP1 peptides.

a *P*-values indicate the significance of differences between groups in Kruskal-Wallis or χ^2^ tests.

BKPyV-specific CD8 T cell functionalities, including specific proliferation, TNFα single production, TNFα and IFNγ double production, and cytotoxicity were stronger in BKPyV-DNAemia KTRs than in bp-BKPyVAN KTRs (Figure 2a and c–h, and Table 2). In contrast and as previously reported in chronic viral replication, single IFNγ production was the last conserved and was similar between BKPyV-DNAemia and BKPyVAN KTRs (Figure 2e and Table 2).^21^ BKPyV-DNAuria KTRs had better BKPyV-specific CD8 proliferation than other KTRs (Figure 2a and c, and Table 2). The functionality of CD8 effectors, that is, coproduction of IFNγ and TNFα and cytotoxicity, was better in BKPyV-DNAuria KTRs as compared to bp-BKPyVAN KTRs, but not comparatively to BKPyV-DNAemia KTRs (Figure 2f and h, and Table 2).

**Figure 3:**
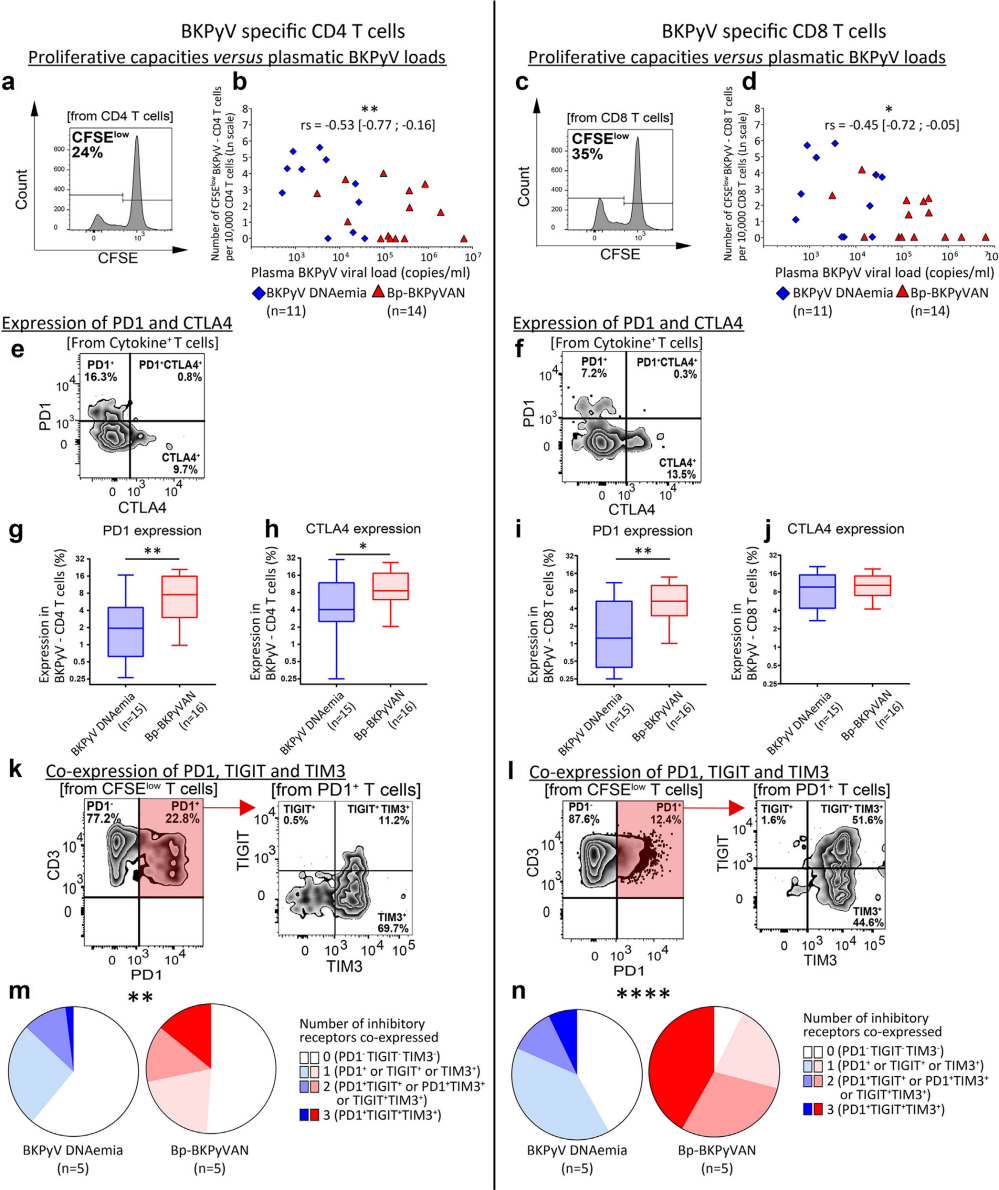
BKPyV-specific CD8 T cell exhaustion in KTRs with biopsy-proven BKPyVAN. We examined the expression of inhibitory receptors on BKPyV-specific CD4 and CD8 T cells in KTR groups as defined above. (a) shows a representative plot of CFSE dilution on CD4 T cells following activation with 2 BKPyV peptide pools (LT-Ag and VP1 peptide pools) for 5 days. (b) shows the correlation between proliferative BKPyV-specific CD4 T cells and plasma BKPyV loads. (c) shows a representative plot of CFSE dilution in CD8 T cells following activation with 2 BKPyV peptide pools for 5 days. (d) shows the correlation between proliferative BKPyV-specific CD8 T cells and plasma BKPyV loads. (e) shows a representative plot of PD1 and CTLA4 expression on IFNγ and/or TNFα producing CD4 T cells following overnight activation with 2 BKPyV peptide pools (Boolean gate analysis). (f) shows a representative plot of PD1 and CTLA4 expression on IFNγ and/or TNFα producing CD8 T cells following overnight activation with 2 BKPyV peptide pools (Boolean gate analysis). (g) and (h) show the expression of PD1 and CTLA4 on BKPyV-specific CD4 T cells, respectively. (i) and **(**j) show the expression of PD1 and CTLA4 on BKPyV-specific CD8 T cells, respectively. (k) shows a representative plot of PD1, TIGIT, and TIM3 coexpression on proliferative BKPyV-specific CD4 T cells (CFSE low cells following activation for 5 days with 2 BKPyV peptide pools). (l) shows a representative plot of PD1, TIGIT and TIM3 coexpression on proliferative BKPyV-specific CD8 T cells (CFSE low cells following activation for 5 days with 2 BKPyV peptide pools). (m) shows the coexpression of PD1, TIM3, and TIGIT on BKPyV-specific CD4 T cells. (n) shows the coexpression of PD1, TIM3, and TIGIT on BKPyV-specific CD8 T cells. CFSE low BKPyV-specific CD4 or CD8 T cells were expressed as the number of cells per 10,000 CD4 or CD8 T cells and then subjected to natural logarithm transformation to normalize the data distributions (b and d). The proportion of patients expressing the inhibitory receptors PD1, TIM3, and TIGIT, were classified as those expressing none (PD1- TIGIT- TIM3-), 1 (PD1^+^ or TIGIT^+^ or TIM3^+^), 2 (PD1^+^ & TIGIT^+^ or PD1^+^ & TIM3^+^ or TIGIT^+^ & TIM3^+^) or 3 (PD1^+^ TIGIT^+^ TIM3^+^) of the inhibitory receptors (m and n). Statistical analysis was performed by using nonparametric Spearman’s correlation (b and d), Mann-Whitney *U* tests (g–j), and χ^2^ tests for trend (m and n). Spearman's rank correlation coefficient and the 95% confidence interval are represented. Box and whiskers plots show the median and (10th–90th percentile). ∗*P* < 0.05, ∗∗*P* < 0.005, ∗∗∗∗*P* < 0.0001. BKPyV, BK polyomavirus; BKPyVAN, BK polyomavirus-associated nephropathy; CTLA4, cytotoxic T lymphocyte-associated protein 4; IFNγ, interferon-γ; KTR, kidney transplant recipient; LT-Ag, large tumor antigen; PBMC, peripheral blood mononuclear cell; PD1, programmed cell death 1; TIGIT, T cell immunoreceptor with Ig and ITIM domains; TIM3, T cell immunoglobulin and mucin domain-containing-3; TNFα, tumor necrosis factor-α; VP1, viral protein-1.

The results show a loss of CD8 functionality in bp-BKPyVAN. This impairment was selective to BKPyV, because CD8 functionality against other recall antigens (CEF) was similar between KTR groups (Table 3 and Supplementary Figure S4) and, thus, could not be directly related to the level or the nature of the therapeutic immunosuppression. This was confirmed by multivariate analysis (Tables 4 and 5) showing that thymoglobulin use was not associated with bp-BKPyVAN (*P* = 0.146), in contrast to BKPyV-specific CD8 T cell proliferation (*P* = 0.009, odds ratio [95% confidence interval] = 0.22 [0.08–0.59]) (Table 5). In addition, a negative correlation was found between the level of BKPyV-specific CD4 and CD8 functionality, that is, proliferative capacities, monoproduction of TNFα and coproduction of IFNγ and TNFα, and the level of plasma BKPyV load (Figure 3a–d and Table 2), whereas bp-BKPyVAN KTRs had the highest plasma BKPyV loads (Figure 1a). Together, our observations point out a defect of BKPyV-specific CD4 and CD8 T cells as a prominent factor for the evolution toward bp-BKPyVAN.

**Table 3:** Comparison of other anti-viral specific T cell functionalities between KTR groups defined on the basis of CEF reactivation

Assessment of antiviral T cell responses	BKPyV-DNAuria	BKPyV-DNAemia	Bp-BKPyVAN	*P*-value^a^
CEF stimulation (n)	10	10	10	
Proliferative capacities: CFSE low CD8 T cells (cell nb)	4.81 (3.64–6.11)	4.22 (0.10–6.13)	3.85 (2.03–5.51)	0.726
Cytokine secretion capacities: IFN^+^ CD8 T cells (cell nb)	2.32 (0.22–3.45)	2.27 (0.33–5.68)	2.25 (1.64–7.59)	0.920
Cytokine secretion capacities: TNF^+^ CD8 T cells (cell nb)	0.03 (0.03–1.44)	0.88 (0.03–2.46)	0.03 (0.03–2.00)	0.711
Cytokine secretion capacities: TNF^+^ & IFN^+^ CD8 T cells (cell nb)	1.31 (0.50–3.90)	1.42 (0.03–15.42)	1.67 (0.03–3.46)	0.928
Cytotoxic capacities: 7AAD^+^ target cells (cell nb)	20 (9.7–28.1)	10.6 (6.9–22.95)	17.4 (3.85–56.9)	0.777

7AAD, 7-Aminoactinomycin D; BKPyV, BK polyomavirus; BKPyV-DNAemia, kidney transplant recipients with plasma BKPyV reactivation; BKPyV-DNAuria, kidney transplant recipients with urinary BKPyV reactivation; Bp-BKPyVAN, kidney transplant recipients with biopsy-proven BKPyV-associated nephropathy; CEF, pool of cytomegalovirus; Epstein Barr virus and influenza virus peptides; cell nb, normalized BKv-specific T-cell to 10,000 T cell corresponding CD4 or CD8 T cells; IFN, interferon; LT-Ag, large tumor antigen; *n*, number of patients; TNF, tumor necrosis factor.

Continuous data are expressed as medians and interquartile ranges.

Correlations were evaluated with the nonparametric Spearman’s rank correlation test.

For each patient, 2 BKPyV-specific responses were analyzed after stimulation with LT-Ag and VP1 peptides.

a *P*-values indicate the significance of differences between groups in Kruskal-Wallis or χ^2^ tests.

**Table 4:** Risk factors associated with BKPyV-DNAemia and biopsy-proven BKPyVAN in the context of BKPyV reactivation-univariate analysis

Risk factors associated with BKPyV-DNAemia and Bp-BKPyVAN in the context of BKPyV reactivation	BKPyV-DNAemia (vs. BKPyV-DNAuria)	*P*-value^a^	Bp-BKPyVAN (vs. BKPyV-DNAuria)	*P*-value^a^	Bp-BKPyVAN (vs. BKPyV-DNAemia)	*P*-value^a^
	OR (95% CI)		OR (95% CI)		OR (95% CI)	
Immunosuppressive treatment						
Thymoglobulin	3.43 (1.03–11.48)	0.068	4.75 (1.41–16.05)	0.036	1.38 (0.45–4.25)	0.569
Antimetabolite exposure	1.12 (1.02–1.23)	0.030	1.17 (1.06–1.30)	0.006	1.05 (1.00–1.10)	0.058
HLA mismatches						
Total number of (HLA-A, -B, -DR,-DQ) mismatches	1.57 (1.13–2.19)	0.024	1.08 (0.81–1.45)	0.605	0.69 (0.50–0.95)	0.036
0 (HLA-DR) mismatch	1 (ref)	0.036	1 (ref)	0.692	1 (ref)	0.070
1 (HLA-DR) mismatch	7.50 (1.29–43.69)		1.67 (0.47–5.93)		0.22 (0.04–1.30)	
2 (HLA-DR) mismatches	15.60 (2.53–96.08)		1.60 (0.37–6.95)		0.10 (0.02–0.63)	
Proliferative BKPyV (LT-Ag and VP1) CD4 T cells						
Response for LT-Ag (cell nb)	0.72 (0.48–1.07)	0.159	0.62 (0.42–0.92)	0.054	0.86 (0.59–1.27)	0.453
Response for VP1 (cell nb)	0.82 (0.56–1.20)	0.298	0.57 (0.38–0.84)	0.015	0.69 (0.47–1.02)	0.093
Proliferative BKPyV (LT-Ag and VP1) CD8 T cells						
Response for LT-Ag (cell nb)	0.58 (0.36–0.92)	0.030	0.39 (0.23–0.66)	0.003	0.67 (0.43–1.05)	0.084
Response for VP1 (cell nb)	0.54 (0.33–0.87)	0.017	0.37 (0.21–0.64)	<0.001	0.68 (0.45–1.04)	0.075

BKPyV, BK polyomavirus; BKPyV-DNAemia, kidney transplant recipients with plasma BKPyV reactivation; BKPyV-DNAuria, kidney transplant recipients with urinary BKPyV reactivation; Bp-BKPyVAN, kidney transplant recipients with biopsy-proven BKPyV-associated nephropathy; cell nb, normalized BKv-specific T-cellT cell frequencies; HLA, human leukocyte antigen; LT-Ag, large tumor antigen; OR (95% CI), odds ratio with 95% confidence interval; VP1, viral protein-1.

*P*-values indicate the significance of differences between groups in:.

a Multinomial univariate logistic regression analyses with correction for false discovery rate.

**Table 5:** Risk factors associated with BKPyV-DNAemia and biopsy-proven BKPyVAN in the context of BKPyV reactivation**-**multivariate analysis

Risk factors associated with BKPyV-DNAemia and Bp-BKPyVAN in the context of BKPyV reactivation	BKPyV-DNAemia (vs. BKPyV-DNAuria)	*P*-value^a^	Bp-BKPyVAN (vs. BKPyV-DNAuria)	*P*-value^a^	Bp-BKPyVAN (vs. BKPyV-DNAemia)	*P*-value^a^
	OR (95% CI)		OR (95% CI)		OR (95% CI)	
Thymoglobulin	69.55 (2.04–2372.54)	0.054	14.38 (0.47–443.08)	0.146	0.21 (0.02–1.73)	0.146
(HLA-DR) mismatches						
0 mismatch	1 (ref)	0.047	1 (ref)	0.194	1 (ref)	0.047
1 mismatch	94.96 (1.25–7188.84)		32.24 (0.75–1393.67)		0.34 (0.02–6.23)	
2 mismatches	458.23 (5.32–39,466.53)		13.52 (0.17–1057.48)		0.03 (0.002–0.53)	
Proliferative BKv-specific CD8 T cell response	0.33 (0.12–0.92)	0.051	0.22 (0.08–0.59)	0.009	0.66 (0.37–1.17)	0.154

BKPyV, BK polyomavirus; BKPyV-DNAemia, kidney transplant recipients with plasma BKPyV reactivation; BKPyV-DNAuria, kidney transplant recipients with urinary BKPyV reactivation; Bp-BKPyVAN, kidney transplant recipients with biopsy-proven BKPyV-associated nephropathy; cell nb, normalized BKv-specific T-cellT cell frequencies; HLA, human leukocyte antigen; OR (95% CI), odds ratio with 95% confidence interval.

*P*-values indicate the significance of differences between groups in:.

a Wald tests (multivariate multinomial logistic regression) with correction for false discovery rate.

### BKPyV-Specific CD8 T Cell Exhaustion in Patients With bp-BKPyVAN

Expression of inhibitory receptors is a major cause of T cell functional defect. As shown in Figure 3, the level of PD1 expression on BKPyV-specific CD4 and CD8 T cells was significantly higher in bp-BKPyVAN KTRs than in other groups (Figure 3e–g and i, and Supplementary Figure S5). In addition, cytotoxic T lymphocyte-associated protein 4 was more frequently expressed in BKPyV-specific CD4 T cells from bp-BKPyVAN KTRs, whereas it was not the case for BKPyV-specific CD8 T cells (Figure 3e, f, h, and j; and Supplementary Figure S5). Coexpression of inhibitory receptors, including PD1, T cell immunoreceptor with Ig and ITIM domains, and T cell immunoglobulin and mucin domain-containing-3, was higher in BKPyV-specific CD4 and CD8 T cells from patients with bp-BKPyVAN (Figure 3k–n, and Supplementary Figure S6). These results were confirmed on BKPyV-specific CD8 T cells isolated using BKPyV-specific pentamers from a subgroup of HLA-A2 or HLA-B7 KTRs (Supplementary Figure S7). These results suggest that the more profound defect of BKPyV-specific CD4 and CD8 T cells observed in bp-BKPyVAN may be related to a higher expression of inhibitory receptors. Together, the poor functionality (Figure 2) and the increased expression of inhibitory receptors (Figure 3) point out an exhaustion phenotype for BKPyV-specific CD4 and CD8 T cells in bp-BKPyVAN KTRs.

### Mismatched Allogeneic CD4 T Cells Restore BKPyV-Specific CD8 T cell Functionality by Allogeneic CD4 Help

**Figure 4:**
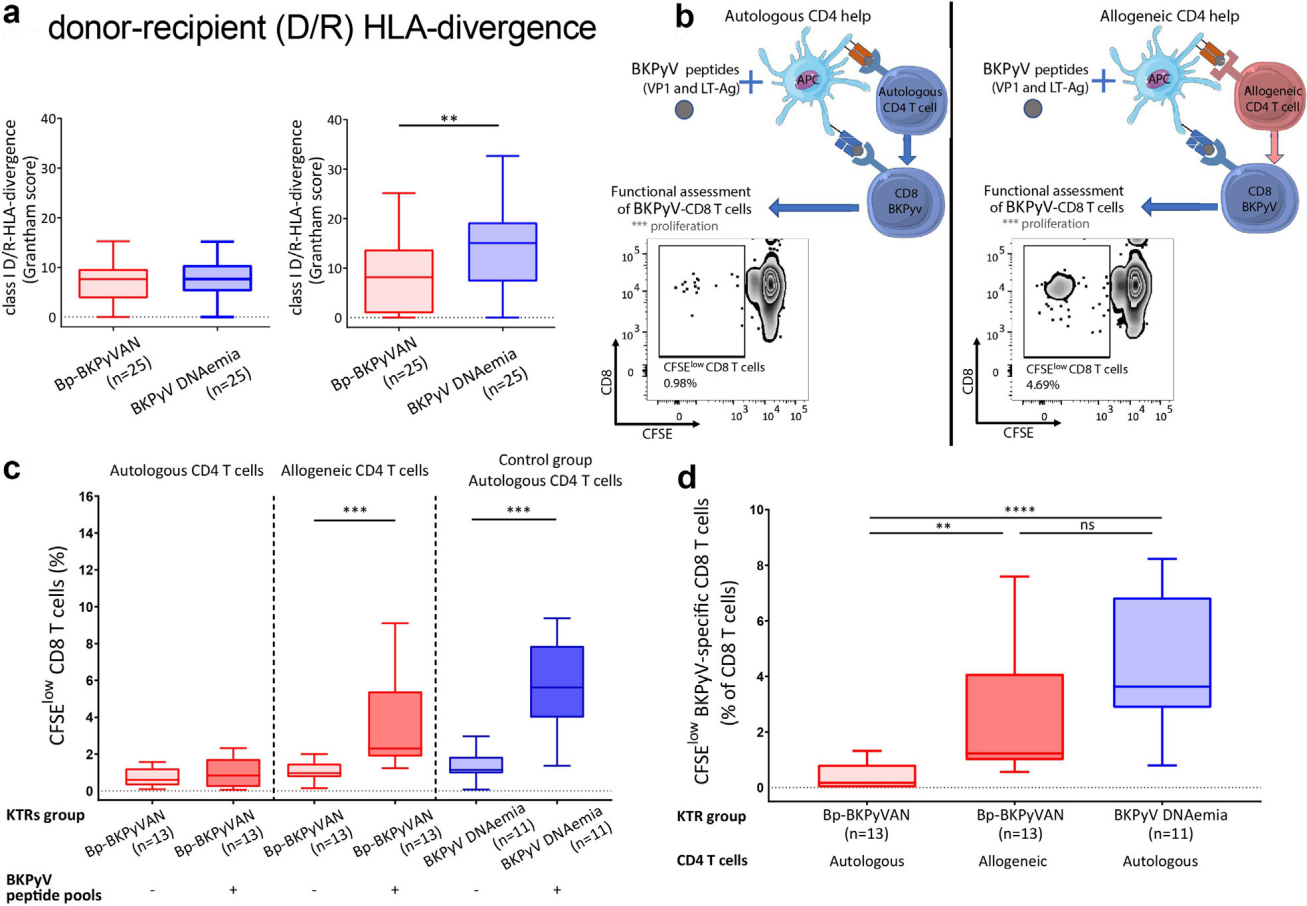
Mismatched allogeneic CD4 T cells restore BKPyV-specific CD8 T cell functionality by allogeneic CD4 help. (a) shows class I and class II D/R-HLA divergence (calculated using the Grantham score) in KTRs with BKPyV-DNAemia and biopsy-proven BKPyVAN. (b) corresponds to the schematic representation of allogeneic CD4 T cell rescue to BKPyV-specific CD8 T cells. (c) shows CD8 proliferation (CFSE dilution) with and without BKPyV peptide pools in the presence of autologous or allogeneic CD4 T cells. Briefly, PBMCs from bp-BKPyVAN KTRs were depleted from CD4 T cells and cultured for 5 days with allogeneic or autologous CD4 T cells and BKPyV peptide pools (Methods). A control group corresponds to PBMCs from BKPyV-DNAemia KTRs treated in autologous condition. (d) shows BKPyV-specific CD8 proliferation in the presence of autologous or allogeneic CD4 T cells in bp-BKPyVAN KTRs and the control group. Results are expressed as delta percentages of CFSE low CD8 T cells between the conditions with and without BKPyV peptide pools. Statistical analysis was performed by using (a) Mann-Whitney *U* tests, (c) Wilcoxon matched-pairs signed-rank tests between autologous and allogeneic comparisons, and (d) Kruskal-Wallis tests followed by Dunn’s multiple comparison tests. ∗∗*P* < 0.005, ∗∗∗*P* < 0.0005, ∗∗∗∗*P* < 0.000. BKPyV, BK polyomavirus; BKPyVAN, BK polyomavirus-associated nephropathy; CD4 BK-v, BKPyV-specific CD4 T cell; CD8 BK-v, BKPyV-specific CD8 T cell; donor APC, donor antigen-presenting cell; D/R-HLA divergence, donor-recipient human leukocyte antigen divergence; KTR, kidney transplant recipient; ns, nonsignificant.

In a multivariate analysis, a higher number of HLA-DR mismatches appears protective against bp-BKPyVAN, because 2 HLA-DR mismatches were associated with an odds ratio (95% confidence interval) of 0.03 (0.002–0.53) (*P* = 0.047, Table 5). This suggests that major histocompatibility complex class II-mediated allogeneicity may lead to a better control of BKPyV replication into the kidney allograft, possibly through a better BKPyV-specific T cell response. In addition to the number of mismatches, the allogeneic potential may be assessed by HLA evolutionary divergence.^22^ HLA evolutionary divergence between an individual’s HLA alleles is a quantitative “pairwise” distance determined by the Grantham score, in which the physiochemical properties of amino acids, and thus the functional similarity are considered.^14^, ^15^, ^16^, ^17^ We determined class I and class II D/R-HLA divergence by calculating the Grantham score^14^ between donor and recipient’s HLA alleles. D/R-HLA divergences strongly correlate with HLA mismatches for class I and class II (Supplementary Figure S8A and B). Moreover, class II D/R-HLA divergence correlates with the percentage of proliferating allogeneic CD4 T cells, whereas this was not the case for class I D/R-HLA divergence (Supplementary Figure S8C and D). A higher D/R-HLA divergence may reflect a higher difference between the immunopeptidomes of donor and recipient and, therefore, a higher allogeneicity. Consistently with the finding that a higher number of HLA-DR mismatches was protective for bp-BKPyVAN, KTRs with bp-BKPyVAN had a significantly lower score for class II D/R-HLA divergence than patients with BKPyV-DNAemia, whereas there was no significant difference for class I D/R-HLA divergence (*P* = 0.0037 and *P* = 0.42, respectively) (Figure 4a). These results suggest that a higher class II D/R-HLA divergence leads to better control of the BKPyV replication in the graft and may protect against bp-BKPyVAN.

**Figure 5:**
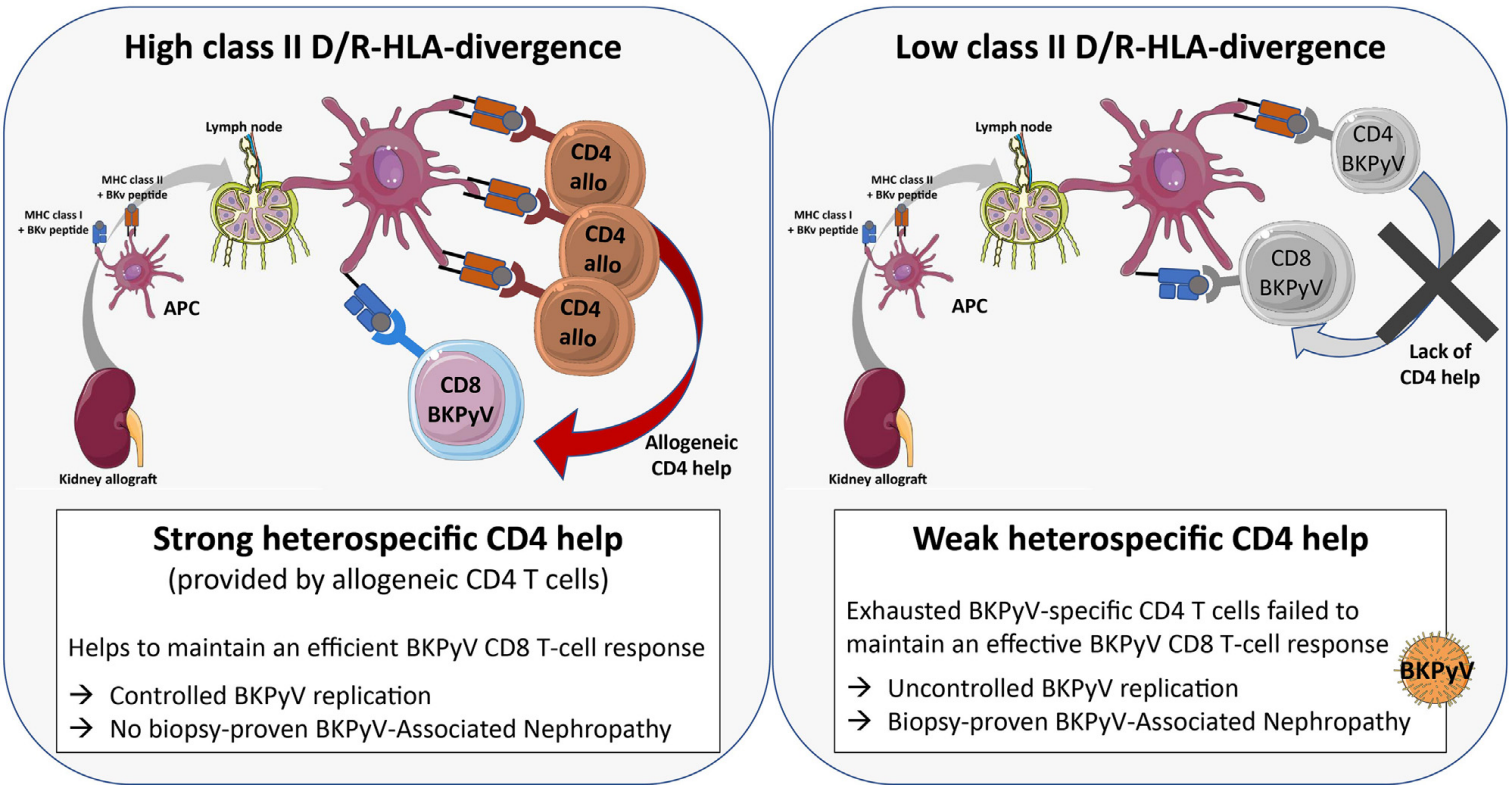
A schematic representation of how high-class II D/R-HLA divergence may improve BKPyV-specific CD8 T cell responses in KTRs. BKPyV, BK polyomavirus; CD4 allo, allogeneic CD4 T cell; CD4 BK-v, BKPyV-specific CD4 T cell; CD8 BK-v, BKPyV-specific CD8 T cell; donor APC, donor antigen-presenting cell; D/R-HLA divergence, donor-recipient human leukocyte antigen divergence; MHC, major histocompatibility complex.

We previously reported that heterospecific CD4 help, provided by CD4 T cells directed to a first antigen, could provide effective help to memory CD8 T cells directed against a second distinct antigen.^23^^,^^24^ Therefore, we examined whether allogeneic CD4 T cells may provide effective help to BKPyV-specific memory CD8 T cells (Figure 4b). In bp-BKPyVAN KTRs, allogeneic CD4 T cells led to the recovery of proliferative capacities of BKPyV-specific memory CD8 T cells (Figure 4c and d). Noteworthy, the recovery of proliferative BKPyV-specific CD8 T cell level was up to the level observed in BKPyV-DNAemia KTRs (Figure 4d). Allogeneic controls without BKPyV-peptides confirmed that CD8 proliferative responses were BKPyV-specific, because CD8 T cell proliferative response significantly increased only in the presence of BKPyV-peptides (*P* = 0.0002). In 5 BKPyV-DNAemia KTRs, we added allogeneic or autologous CD4 T cells to CD4-depleted PBMCs. In contrast to bp-BKPyVAN KTRs, allogeneic CD4 T cells did not significantly improve the BKPyV-specific proliferation of CD8 T cells from BKPyV-DNAemia KTRs as compared to autologous CD4 T cells (data not shown, *P* = 0.19, *n* = 5). Altogether, our results indicate that a higher class II D/R-HLA divergence, which may lead to stronger allogeneic CD4 T cell response, is associated with better BKPyV-specific memory CD8 T cell response and may protect against BKPyVAN. A possible underlying mechanism is providing more effective help to memory BKPyV-specific CD8 T cells by allogeneic CD4 T cells than by BKPyV-specific CD4 T cells that exhibit hallmarks of exhaustion (Figure 5).

## Discussion

Bp-BKPyVAN in KTR is associated with a higher plasma BKPyV load and poor graft function. Our results suggest that the loss of functional BKPyV-specific T cell responses during bp-BKPyVAN involves immune exhaustion. We also provide evidence that a high level of class II D/R-HLA divergence may protect against bp-BKPyVAN by providing allogeneic CD4 help that sustains BKPyV-specific CD8 T cell responses, prevents exhaustion, and allows better control of BKPyV replication.

BKPyV infection is a significant threat in kidney transplantation, which may lead to BKPyAN and graft loss. Because of the absence of specific treatment, reducing immunosuppression is the primary intervention for biopsy-proven and presumptive-BKPyVAN. Despite this current practice, we show that lowering it cannot prevent the high rate of graft loss. Immunosuppressive regimens should, therefore, be adapted earlier following the detection of BKPyV-DNAemia in case of possible or presumptive-BKPyVAN. However, the decrease of immunosuppression is associated with an increased risk of acute cellular rejection in up to 20% of cases.^8^ Therefore, achieving an optimal balance between the control of BKPyV replication and the risk of acute graft rejection following the adaptation of immunosuppressive therapy remains a major challenge.

Several immunosuppressive strategies have been identified as critical factors in BKPyV reactivation.^25^ Thymoglobulin, a polyclonal antibody or alemtuzumab induction therapy that causes profound T cell depletion, has been associated with BKPyV reactivation.^26^ In our study, thymoglobulin therapy was not an independent risk factor for bp-BKPyVAN occurrence. This can be due to the number of patients enrolled and treated by depleting agents in the study. However, a statistical trend was observed for BKPyV-DNAemia, suggesting that thymoglobulin use may favor BKPyV-DNAemia but is not sufficient to generate bp-BKPyVAN.

BKPyV-specific T cell response plays a crucial role in controlling BKPyV replication.^27^ BKPyV-specific 9mer CD8 T cell responses have been shown to correlate with the clearance of BKPyV-DNAemia.^28^ After kidney transplantation, its presence has been shown to be predictive of an absence or a low level of BKPyV-DNAemia.^29^ In addition, an expansion of BKPyV-specific T cells was observed after immunosuppression reduction and was associated with virus clearance.^30^ The determination of the BKPyV-specific T cell function would help to distinguish patients with uncontrolled BKPyV-DNAemia, who would benefit from a reduction of immunosuppression, from those with self-limiting BKPyV-DNAemia who will be exposed to a risk of rejection because of unnecessary immunosuppression reduction.^31^ Conversely, the reappearance of BKPyV-specific T cell functions will help physicians to identify an effective status of BKPyV-specific T cells and to reincrease, if necessary, immunosuppression in order to reduce the subsequent risk of rejection.

In healthy individuals, BKPyV-specific T cells are polyfunctional and control the BKPyV replication.^32^ Conversely, a loss of functionality is observed after kidney transplantation.^33^^,^^34^ Sustained chronic viral replication may trigger BKPyV-specific T cell exhaustion that worsens T cell functionality, leading to uncontrolled BKPyV replication in KTRs and then to bp-BKPyVAN. Although either the expression of PD1 or T cell immunoglobulin and mucin domain-containing-3 on BKV-specific CD4 T cells has been shown in the context of sustained BKPyV-DNAemia,^35^ we demonstrate here an exhausted-like phenotype of BKPyV-specific CD8 T cells in the context of bp-BKPyVAN, because these cells had a state of hyporesponsiveness associated with a high level and coexpression of inhibitory receptor expression.^36^, ^37^, ^38^ Interestingly, this phenotype was observed for BKPyV-specific T cells but not for other virus-specific T cells (i.e., cytomegalovirus, Epstein Barr Virus, and influenza), suggesting a local specific effect on the T cell responses not driven by a general overimmunosuppression.

HLA mismatches favor the development of allogeneic T cell responses that negatively impact allograft survival in KTRs.^39^^,^^40^ In liver transplantation, class I donor-HLA divergence reflecting graft immunogenicity predicts graft rejection independently of the number of donor-recipient HLA mismatches.^22^ In contrast, we found that a high number of HLA-DR mismatches and a higher level of class II D/R-HLA divergence, but not for class I D/R-HLA divergence, may be protective against bp-BKPyVAN, thereby highlighting the impact of allogeneic CD4 T cell response. This is in agreement with previous studies showing that a better HLA matching is associated with a higher frequency of BKPyVAN,^4^^,^^41^^,^^42^ whereas few studies, including patients treated with monoclonal antibodies (OKT3, alemtuzumab, etc.) and facing a substantial number of treated acute rejections reported HLA mismatches as risk factors of BKPyVAN.^43^ Moreover, HLA divergence after allogeneic hematopoietic stem cell transplantation has been shown to be positively associated with better overall survival and relapse-free survival, possibly via graft-versus-leukemia responses.^44^^,^^45^ Here, we provide the first description of the impact of class II D/R-HLA divergence on the ability to control BKPyV infection after kidney transplantation and, in fine, how it may protect against bp-BKPyVAN.

One possibility is that allogeneic CD4 T cell response may provide some help to BKPyV-specific-memory CD8 T cells, improving their functionality. Helping signals from CD4 T cells are critical for optimal maintenance and reactivation of memory CD8 T cell responses.^46^ An *in-vitro* model of expanded BKPyV-specific CD8 T cells, obtained from healthy donors, interacting with autologous monocyte-derived-dendritic cells and autologous-CD4 T cells, lead to reinforce the functionality of BKPyV-specific CD8 T cells.^47^

The CD4-help process between CD4 T cells and CD8 T cells involves simultaneous or sequential interactions with the identical or distinct antigen-presenting cell.^23^^,^^24^ In *in vivo* mice experimental models, we have previously shown that those interactions do not necessarily require recognizing peptides from the same antigenic source, for example, the same pathogen, for helping to be provided.^23^^,^^24^ Here, we show *in vitro* that, allogeneic CD4 T cells from patients with BKPyVAN may rescue BKPyV-specific CD8 T cells in patients with bp-BKPyVAN. This points out that, though allogeneic-CD4 T cells may also be subject to some levels of exhaustion,^48^^,^^49^ they may maintain a higher functionality than BKPyV-specific CD4 T cells in KTRs with sustained BKPyV replication due to the strength of direct allorecognition. The results suggest a double CD4-help of allogeneic-CD4 T cells to both allogeneic-CD8 T cells and BKPyV-specific CD8 T cells following interaction with donor antigen-presenting cells coming from BKPyV-infected kidney allograft. BKPyV-DNAemia usually occurs early following kidney transplantation. Help from allogeneic-CD4 T cells provided during the donor antigen-presenting cell persistence period may be critical for optimal reactivation of BKPyV-specific memory CD8 T cells and may occur during immunosuppression reduction.

Altogether, our results unravel the immunological mechanisms that drive the evolution of BKPyV reactivation toward bp-BKPyVAN. The leading role of allogeneic CD4-help, which modulates the functionality of BKPyV-specific CD8 T cell responses, could pave the way for an *ex vivo* cell therapy approach to rescue recipient BKPyV-specific CD8 T cells. By using monocyte-derived dendritic cells from the recipient loaded with BKPyV peptides and allogeneic CD4 T cells to provide efficient help, sorted recipient BKPyV-specific CD8 T cells could be “resuscitated” and then reinjected to the recipient. The newly known impact of class II D/R-HLA divergence on the control of BKPyV-DNAemia and the evolution toward bp-BKPyVAN may better help to stratify the risk of BKPyVAN and to better tune the level of therapeutic immunosuppression.

## Disclosure

All the authors declared no competing interests.

## Supplementary Material

Supplementary File (PDF) **Supplementary Methods. Figure S1.** Assessment of T cell functionality in terms of proliferative, cytokine production and cytotoxic capacities. **Figure S2.** Assessment of the expression of inhibitory receptors on BKPyV-specific CD4 and CD8 T cells. **Figure S3.** Cytokine production capacities of BKPyV-specific CD4 T cells in KTR groups. **Figure S4.** Polyfunctionality of antiviral CD8 T cells (after CEF activation) in KTR groups. **Figure S5.** Assessment of PD-1 and CTLA4 expression on BKPyV-specific T cells in the different groups of BKPyV reactivation. **Figure S6.** Assessment of inhibitory receptor expression on BKPyV-specific T cells in the BKPyV-DNAemia and bp-BKPyVAN groups. **Figure S7.** Isolation of BKPyV-specific CD8 T cells by BKPyV-specific pentamers from a subgroup of HLA-A2 or HLA-B7 KTRs. **Figure S8.** HLA divergence and allogeneic T cell proliferation. **Table S1.** Therapeutic management before and after bp-BKPyVAN diagnosis. **Table S2.** Assessment of BKPyV and CEF-specific T cell responses in the context of kidney transplantation (KTRs without BKPyV reactivation).

## Footnotes

Supplementary File (PDF)

**Supplementary Methods.**
**Figure S1.** Assessment of T cell functionality in terms of proliferative, cytokine production and cytotoxic capacities. **Figure S2.** Assessment of the expression of inhibitory receptors on BKPyV-specific CD4 and CD8 T cells. **Figure S3.** Cytokine production capacities of BKPyV-specific CD4 T cells in KTR groups. **Figure S4.** Polyfunctionality of antiviral CD8 T cells (after CEF activation) in KTR groups. **Figure S5.** Assessment of PD-1 and CTLA4 expression on BKPyV-specific T cells in the different groups of BKPyV reactivation. **Figure S6.** Assessment of inhibitory receptor expression on BKPyV-specific T cells in the BKPyV-DNAemia and bp-BKPyVAN groups. **Figure S7.** Isolation of BKPyV-specific CD8 T cells by BKPyV-specific pentamers from a subgroup of HLA-A2 or HLA-B7 KTRs. **Figure S8.** HLA divergence and allogeneic T cell proliferation. **Table S1.** Therapeutic management before and after bp-BKPyVAN diagnosis. **Table S2.** Assessment of BKPyV and CEF-specific T cell responses in the context of kidney transplantation (KTRs without BKPyV reactivation).
