# Supplementary material for: Allogeneic CD4 T Cells Sustain Effective BK Polyomavirus-Specific CD8 T Cell Response in Kidney Transplant Recipients
Source: Kidney Int Rep. 2024 May 7;9(8):2498–513. doi: 10.1016/j.ekir.2024.04.070 (PMC11328547; doi:10.1016/j.ekir.2024.04.070)
Supplement: Supplementary File (PDF) — Supplementary Methods. Figure S1. Assessment of T cell functionality in terms of proliferative, cytokine production and cytotoxic capacities. Figure S2. Assessment of the expression of inhibitory receptors on BKPyV-specific CD4 and CD8 T cells. Figure S3. Cytokine production capacities of BKPyV-specific CD4 T cells in KTR groups. Figure S4. Polyfunctionality of antiviral CD8 T cells (after CEF activation) in KTR groups. Figure S5. Assessment of PD-1 and CTLA4 expression on BKPyV-specific T cells in the different groups of BKPyV reactivation. Figure S6. Assessment of inhibitory receptor expression on BKPyV-specific T cells in the BKPyV-DNAemia and bp-BKPyVAN groups. Figure S7. Isolation of BKPyV-specific CD8 T cells by BKPyV-specific pentamers from a subgroup of HLA-A2 or HLA-B7 KTRs. Figure S8. HLA divergence and allogeneic T cell proliferation. Table S1. Therapeutic management before and after bp-BKPyVAN diagnosis. Table S2. Assessment of BKPyV and CEF-specific T cell responses in the context of kidney transplantation (KTRs without BKPyV reactivation). [file mmc1.pdf]

SUPPLEMENTARY MATERIAL – Figure S1. Assessment of T-cell functionality in terms of proliferative, cytokine production and cytotoxic capacities

(a) We assessed lymphocyte functionality by measuring proliferative, cytokine production, and cytotoxic capacities by multicolor flow cytometry (BD LSRFortessa™). Peripheral blood mononuclear cells were incubated alone (unstimulated cells) or with overlapping BKPyV (VP1 or LT-Ag) peptide pools, CEF peptide pools, or with staphylococcal enterotoxin B (SEB). The frequency of responsive T cells was calculated by subtracting the frequency of cells detected in the unstimulated controls.

Proliferative capacity. Lymphocyte proliferation was measured by carboxyfluorescein diacetate succinimidyl ester (CFSE) dilution. Numbers indicate the percentage of $CFSE^{low}$ cells among CD4 and CD8 T cells.

Cytokine production capacity. The production of interferon-γ (IFNγ) and tumor necrosis factor-α (TNFα) was evaluated by intracellular staining. Numbers indicate the percentages of CD4 or CD8 T cells secreting IFNγ, TNFα, or IFNγ & TNFα.

Cytotoxic capacity. BKPyV-specific CD8 cytotoxicity was measured as the number of dead autologous target cells (CD8-depleted PBMC loaded with BKPyV peptides), identified by 7-amino-actinomycin D (7AAD) staining. Numbers indicate the percentage of $7AAD^{+}$ target cells.

7AAD: 7-amino-actinomycin D; BKPyV: BK polyomavirus; CEF: cytomegalovirus, Epstein-Barr virus, and influenza virus peptide pools; CFSE: carboxyfluorescein diacetate succinimidyl ester; IFNγ: interferon-γ; TNFα: tumor necrosis factor-α.

(b) BKPyV-specific T-cell functionality was evaluated in KTRs without BKPyV reactivation.

SUPPLEMENTARY MATERIAL – Figure S2. Assessment of the expression of inhibitory receptors on BKPyV-specific CD4 and CD8 T cells

We evaluated the expression of lymphocyte inhibitory receptors (programmed cell death 1 – PD1, cytotoxic T-lymphocyte-associated protein 4 – CTLA4, T cell immunoreceptor with Ig and ITIM domains – TIGIT, T-cell immunoglobulin and mucin-domain containing-3 – TIM3). Cytokine-secreting BKPyV-specific CD4 and CD8 T cells (Boolean gate from IFNγ- and/or TNFα-secreting cells) were assessed for the expression of PD1 and/or CTLA4 and compared to non-responding T cells (without cytokine production). To evaluate the expression of lymphocyte inhibitory receptors (PD1 and CTLA4), PBMCs were stained with the following antibodies (PD1-BV650, Biotin CTLA4-BV711 Streptavidin).

Proliferative BKPyV-specific CD4 and CD8 T cells were assessed for the expression of PD1 and/or TIM3 and/or TIGIT and compared to non-responding T cells (non-proliferative T cells). To evaluate the expression of lymphocyte inhibitory receptors (PD1, TIM3, and TIGIT), PBMCs were stained with the following antibodies (PD1-PeVio770, TIGIT-PeVio615, and TIM3-APC).

SUPPLEMENTARY MATERIAL – Figure S3. Cytokine production capacities of BKPyV-specific CD4 T cells in KTR groups

BKPyV-specific CD4 T cells were expressed as the number of cells per 10,000 CD4 T cells and then subjected to square root transformation to normalize the data distributions. (n) represents the number of patients. Two BKPyV-specific responses were analyzed for each patient after activation with two BKPyV-specific peptide pools (LT-Ag and VP1-peptides).

BKPyV-DNuria: patients with BKPyV reactivation in urine; BKPyV-DNAemia: patients with plasma BKPyV reactivation; biopsy-proven BKPyVAN: patients with biopsy-proven BK polyomavirus-associated nephropathy; BKPyV: BK polyomavirus; IFNγ: Interferon-γ, LT-Ag: large tumor antigen; *n*: number of patients; TNFα: Tumor necrosis factor-α, TNFα & IFNγ: Co-production of Interferon-γ and Tumor necrosis factor-α, VP1: viral protein 1.
p-values indicate the significance of differences between groups in Kruskal-Wallis tests, followed by Dunn's tests for multiple comparisons. Scatter dot plots are shown with the median and interquartile range. $*p < 0.05$.

SUPPLEMENTARY MATERIAL – Figure S4. Polyfunctionality of antiviral CD8 T cells (after CEF activation) in KTR groups

CD8 T-cell functionality after CEF activation in the groups of KTRs, with an assessment of **(a)** proliferation, **(b)** cytotoxic activity, **(c)** IFNγ production, **(d)** TNFα production, and **(e)** co-production of IFNγ and TNFα.

CEF-specific CD8 T cells were expressed as the number of cells per 10,000 CD8 T cells and then subjected to natural logarithm or square root transformation to normalize the data distributions. (n) represents the number of patients.

BKPyV-DNAuria: patients with BKPyV reactivation in urine; BKPyV-DNAemia: patients with plasma BKPyV reactivation; biopsy-proven BKPyVAN: patients with biopsy-proven BK polyomavirus-associated nephropathy; 7AAD: 7-amino-actinomycin D; CEF: cytomegalovirus, Epstein-Barr virus, and influenza virus peptide pools; CFSE: carboxyfluorescein diacetate succinimidyl ester; IFNγ: interferon-γ; *n*: number of patients; *p*: *p*-values; TNFα: tumor necrosis factor-α.

p-values indicate the significance of differences between groups in Kruskal-Wallis tests. Scatter dot plots are shown, with the median and interquartile range. ns = non-significant.

SUPPLEMENTARY MATERIAL – Figure S5. Assessment of PD-1 and CTLA4 expression on BKPyV-specific T cells in the different groups of BKPyV reactivation

The expression of inhibitory receptors (PD1, CTLA4) was evaluated on BKPyV-specific T cells in patients without BKPyV reactivation, BKPyV-DNAuria, BKPyV-DNAemia and bp-BKPyVAN groups, on CD 4 and CD8 T-cells.

p-values indicate the significance of differences between groups in Mann-Whitney *U* tests. Scatter dot plots are shown with the median and interquartile range. *$p < 0.05$, **$p < 0.005$, ****$p < 0.0001$, n= number of patients.

SUPPLEMENTARY MATERIAL – Figure S6. Assessment of inhibitory receptor expression on BKPyV-specific T cells in the BKPyV-DNAemia and bp-BKPyVAN groups

The expression of inhibitory receptors (PD1, TIM3, and TIGIT) was evaluated on BKPyV-specific T cells in the BKPyV-DNAemia and bp-BKPyVANgroups, as the proportion of patients expressing inhibitory receptors classified as expressing none ($PD1^-$ $TIGIT^-$ $TIM3^-$), one ($PD1^+$ or $TIGIT^+$ or $TIM3^+$), two ($PD1^+$ & $TIGIT^+$ or $PD1^+$ & $TIM3^+$ or $TIGIT^+$ & $TIM3^+$) or three ($PD1^+$ $TIGIT^+$ $TIM3^+$) inhibitory receptors.

BKPyV-DNAuria: patients with BKPyV reactivation in urine; BKPyV-DNAemia: patients with plasma BKPyV reactivation; biopsy-proven BKPyVAN: patients with biopsy-proven BK polyomavirus-associated nephropathy; Inh.R: inhibitory receptors; n: number of patients.

p-values indicate the significance of differences between groups in Mann-Whitney *U* tests. Scatter dot plots are shown with the median and interquartile range. *$p < 0.05$, ns = non-significant.

SUPPLEMENTARY MATERIAL – Figure S7. Isolation of BKPyV-specific CD8 T cells by BKPyV-specific pentamers from a subgroup of HLA-A2 or HLA-B7 KTRs

a- Comparison of identifying BKPyV-specific CD8 T cells (% of CD8 T cells) according to CFSE dilution or using BKPyV-specific pentamers (n=8). **b**- Functional assessment of BKPyV-pentamers positive CD8 T cells: proliferation capacity (CFSE dilution) (n=5) or cytokine production (IFNγ or TNFα) (n=2). **c**- Expression of PD1 and TIGIT in BKPyV-specific CD8 T cells stimulated with BKPyV peptides and identified by BKPyV-specific pentamers. The FASC gating strategy and a representative dot plot of the expression of TIGIT and PD1 in CD8 T-cells from a patient (HLA-A2) with a BKPyVAN are shown. The left panel shows the expression of TIGIT and PD1 in BKPyV-pentamers positive CD8, and the right panel shows their expression on BKPyV-pentamers negative CD8 T cells. **d** shows the percentage of TIGIT or PD1 expression on BKPyV-specific CD8 T cells identified by BKPyV-specific pentamers from 5 patients (HLA-A2 and/or HLA-B7) with a BKPyVAN. PD1 and TIGIT were more frequently observed in BKPyV pentamers positive CD8 T cells than in non-specific CD8 T cells.

ns non-significant, * $p<0.05$.

SUPPLEMENTARY MATERIAL – Figure S8. HLA divergence and allogeneic T cell proliferation.

a and b: Correlation between class I D/R-HLA-divergence and class I HLA mismatches classified as 0, 1, or 2 mismatches in the BKv-viremia and BKvAN groups (a). Correlation between class II D/R-HLA-divergence and class II HLA mismatches classified as 0, 1, or 2 mismatches in the BKv-viremia and BKvAN groups (b). nb: number of class I or II HLA mismatches. **c and d**: Correlation between D/R-HLA-divergence and allogeneic T-cell proliferation. Allogeneic CD4 T cells were stained with CFSE and mixed with allogeneic PBMC depleted from CD4 T cells using anti-CD4 magnetic beads (Miltenyi). Proliferating T cells were evaluated by CFSE dilution at day 5. D/R-HLA-divergence (Grantham score) was calculated for each combination of donor and recipient and each HLA loci. The Grantham score (class I (HLA-A and HLA-B) or II (HLA-DR and HLA-DQ)) was compared to the square root of the percentage of proliferating CD4 T cells. D/R Class-II HLA divergence significantly correlates with the proliferation of allogeneic CD4 T cells but not with D/R Class-I HLA divergence.

**** $p<0.0001$

SUPPLEMENTARY MATERIAL – Table S1. Therapeutic management before and after bp-BKPyVANdiagnosis

SUPPLEMENTARY MATERIAL – Table S2. Assessment of BKPyV and CEF-specific T-cell responses in the context of kidney transplantation (KTRs without BKPyV reactivation)

Supplementary data: Figure S1

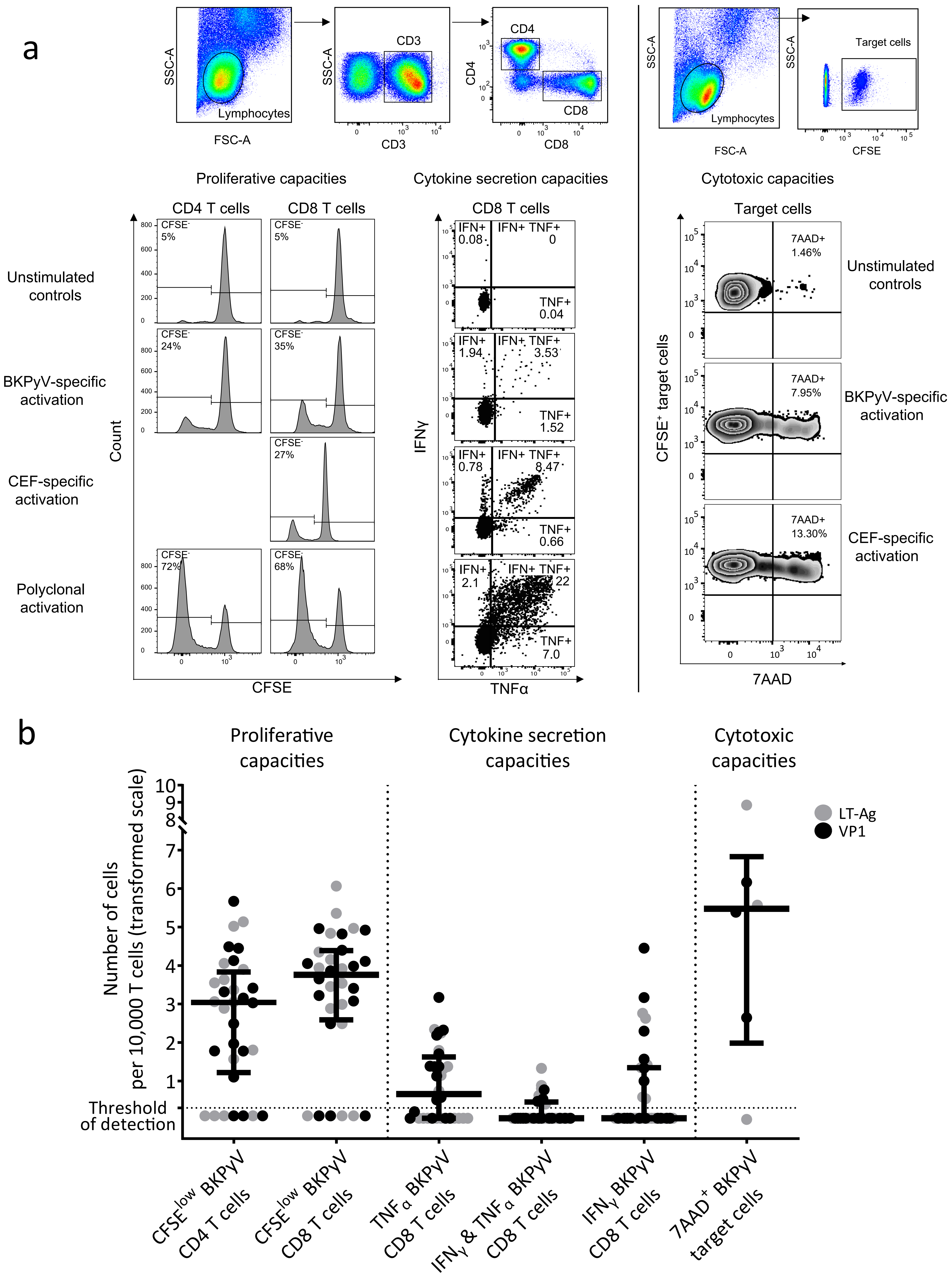

Supplementary data: Figure S2

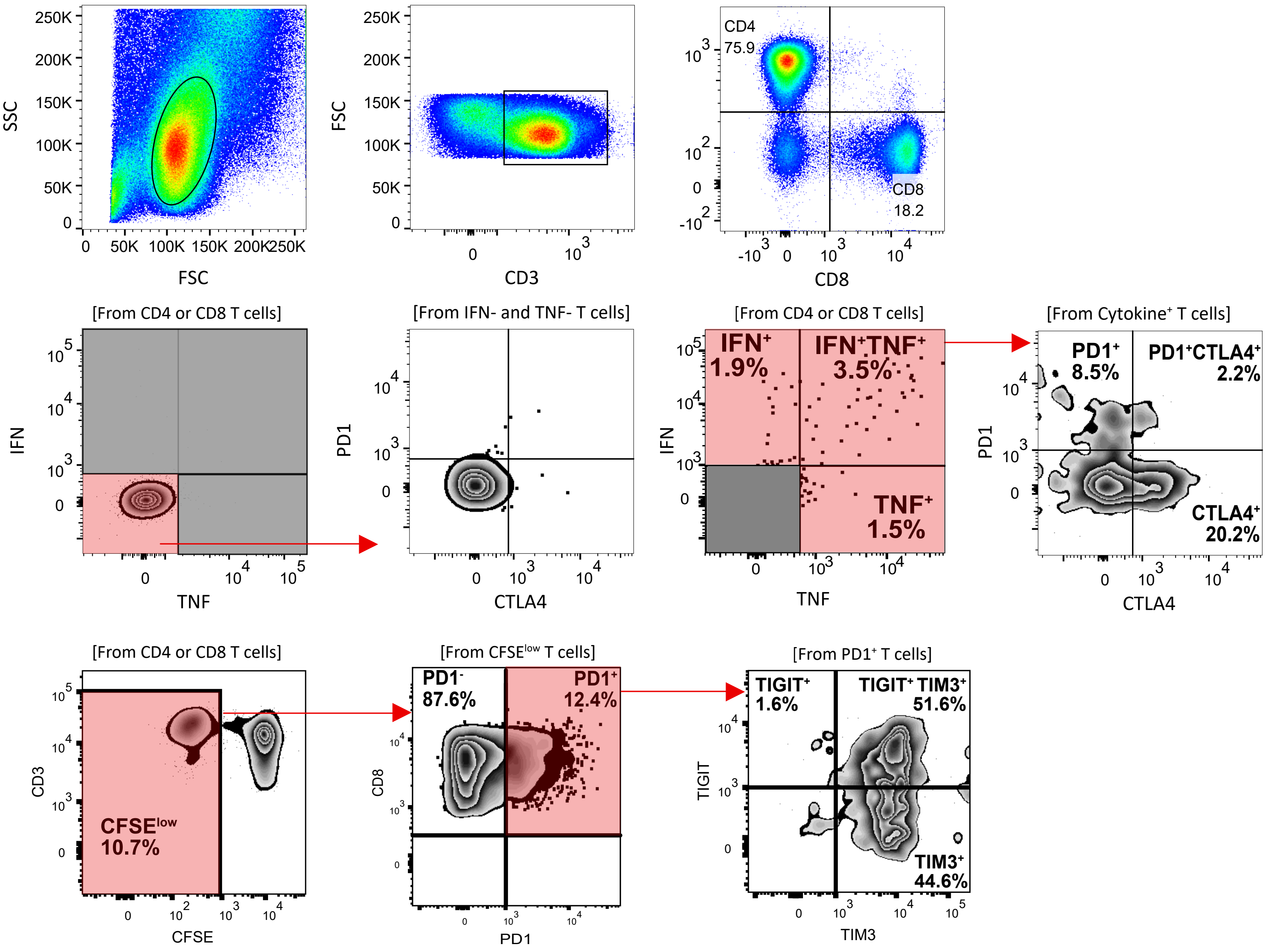

Supplementary data: Figure S3

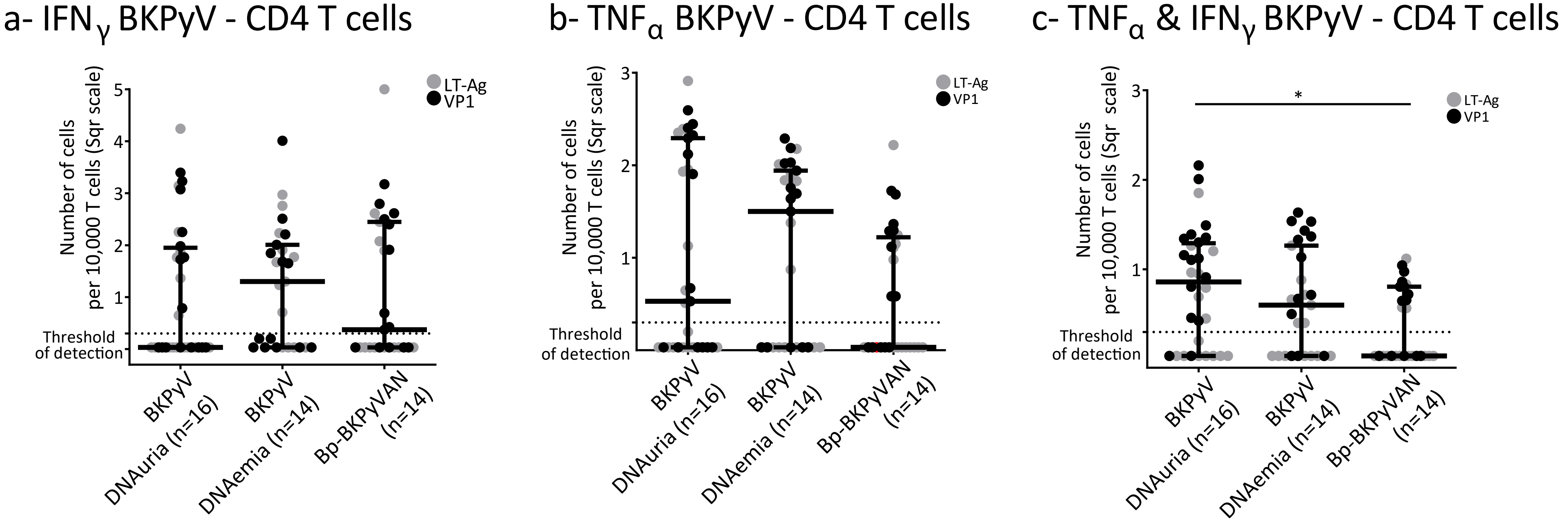

Supplementary data : Figure S4

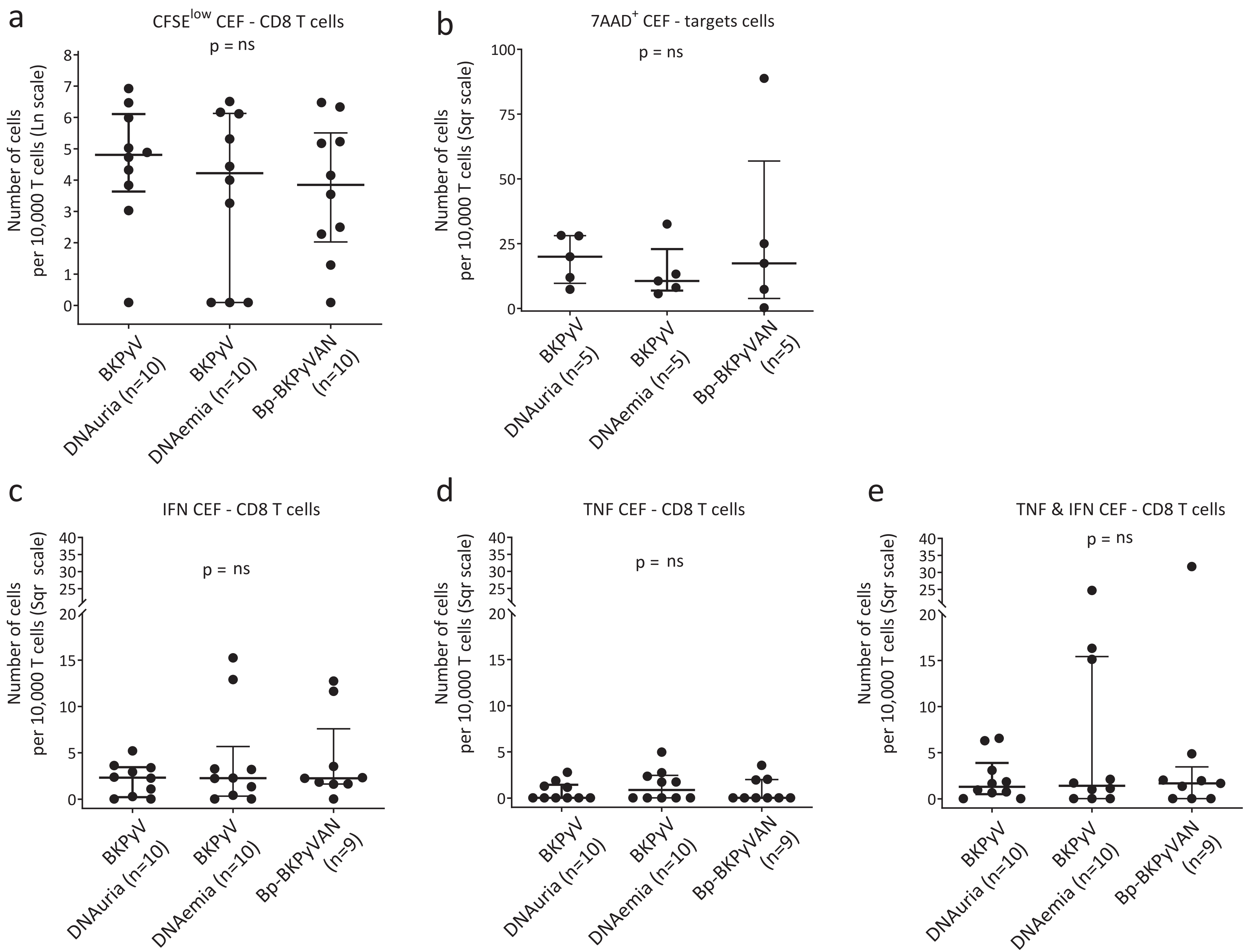

Supplementary data: Figure S5

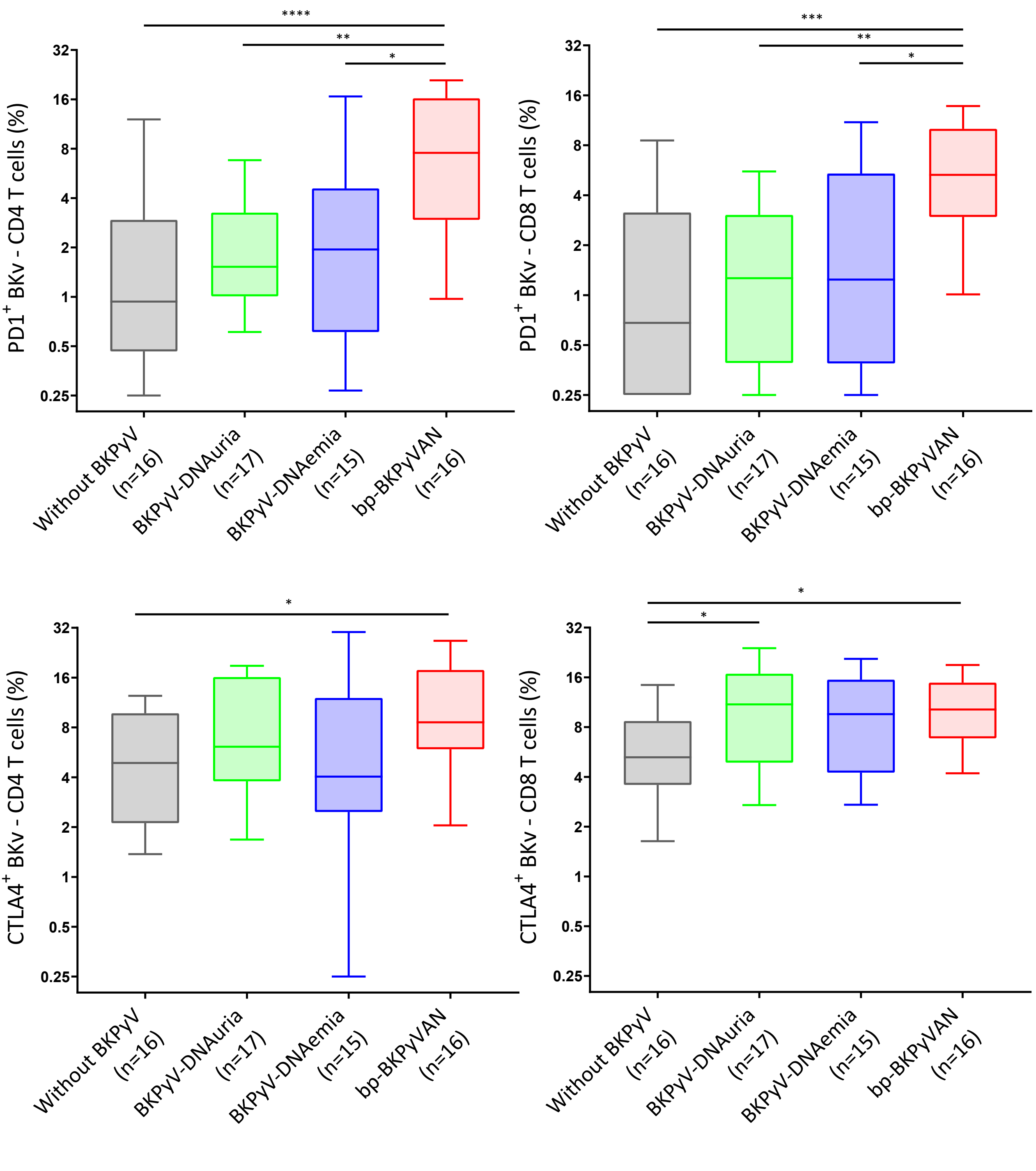

Supplementary data: Figure S6

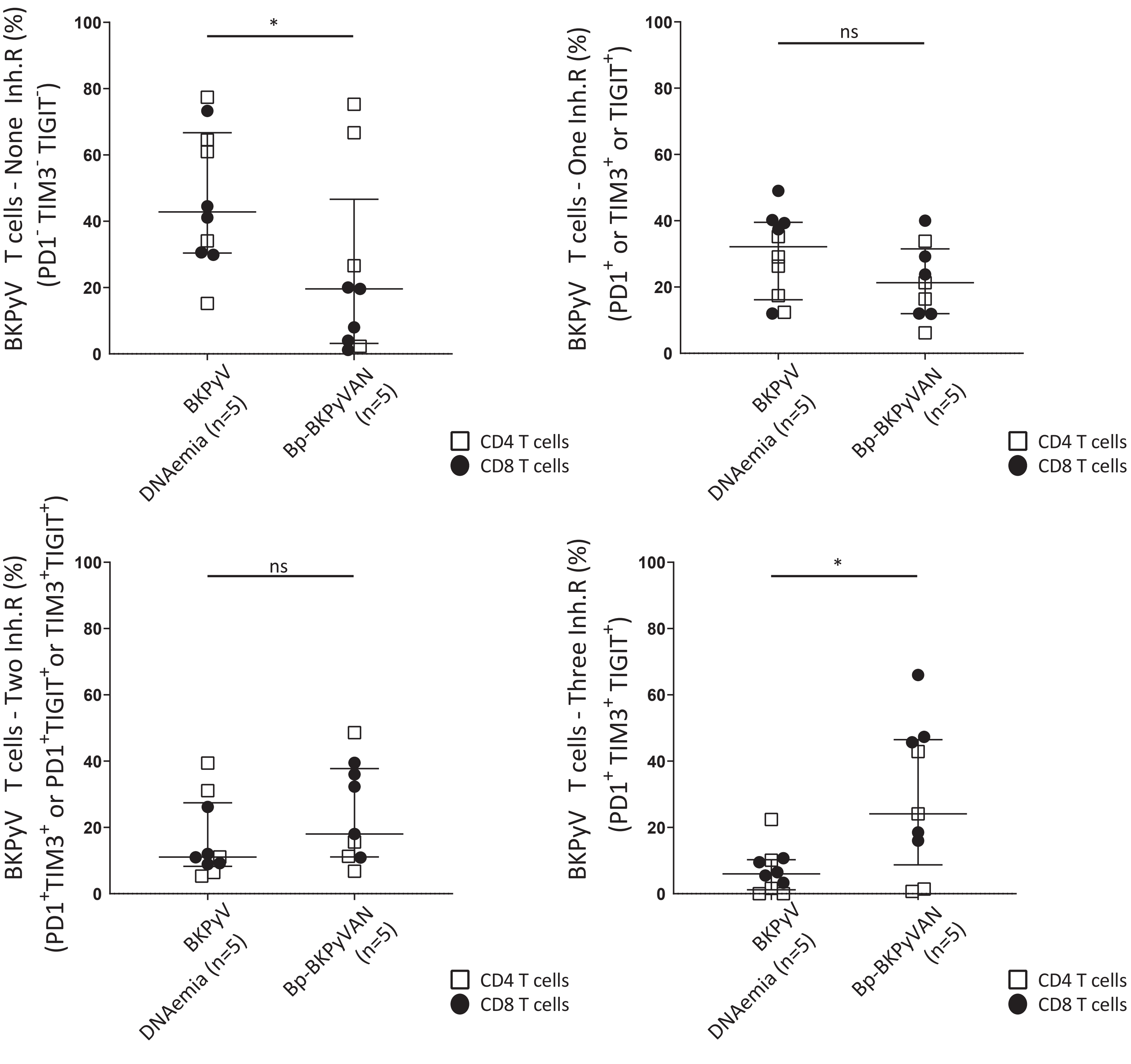

Supplementary data: Figure S7

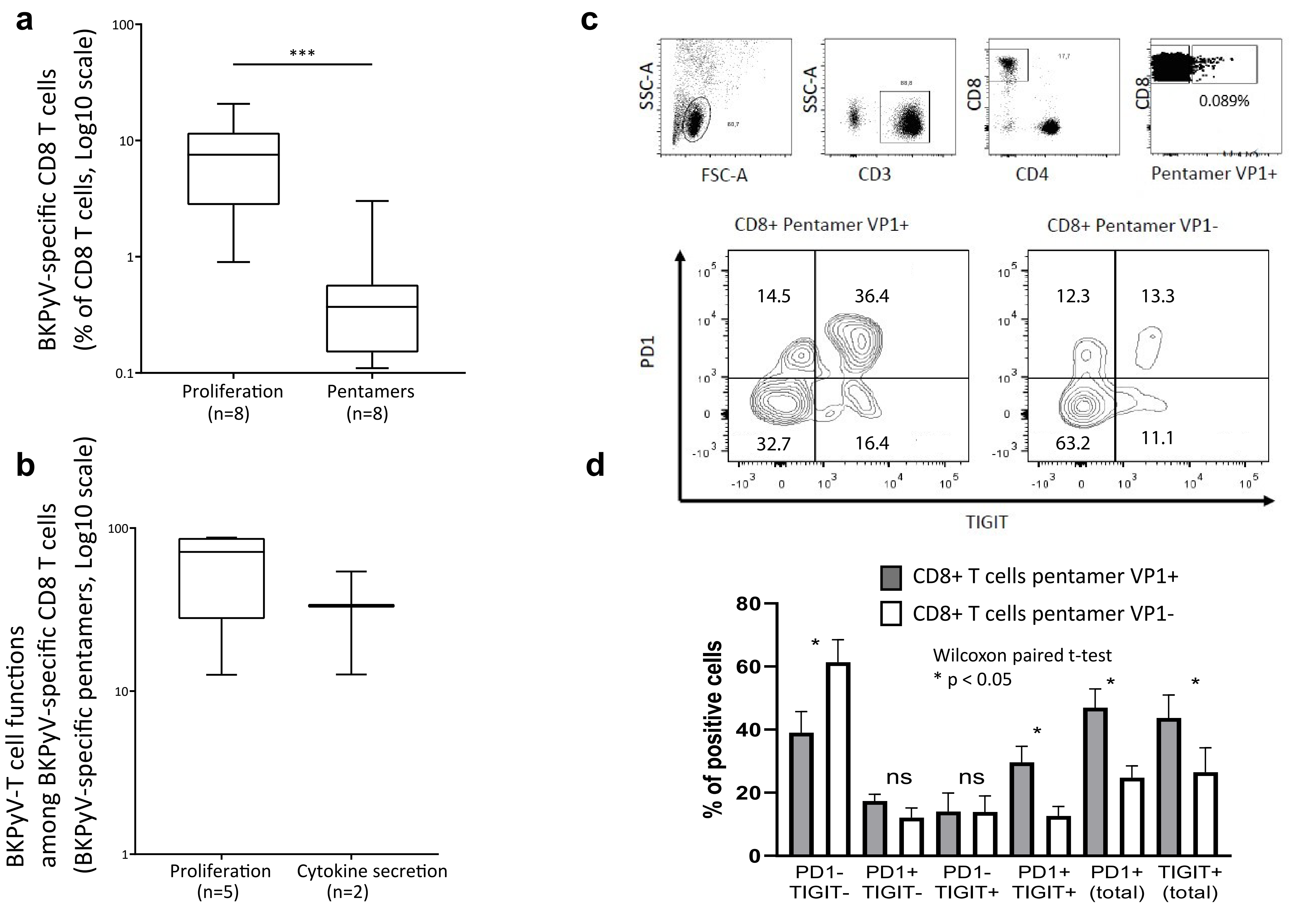

Supplementary data: Figure S8

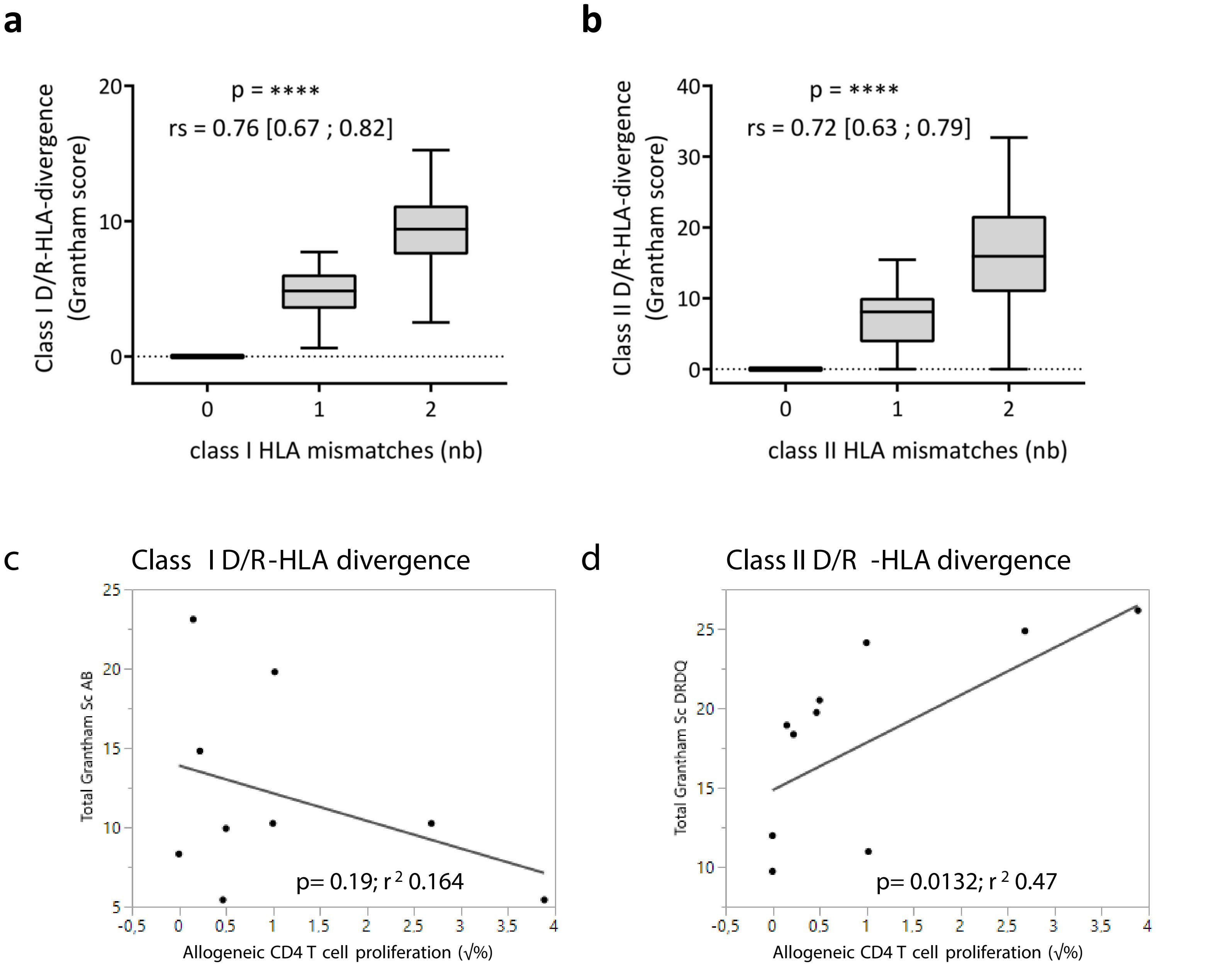

Supplementary data: Table-S1. Therapeutic management before and after bp- BKPyVAN diagnosis

**Therapeutic management in the bp-BKPyVAN group (*n*=25)**	Before bp-BKPyVAN diagnosis	After bp-BKPyVAN diagnosis	p^{i}
Tacrolimus, *n* (%)	21 (84)	18 (72)	0.496
Levels of tacrolimus (ng/mL)	8.7 [6.8-10.4]	5.6 [4.7-6.5]	***<0.0001***
Antimetabolites, *n* (%)	10 (40)	6 (24)	0.364
Everolimus, *n* (%)	5 (20)	9 (36)	0.345

Bp-BKPyVAN: biopsy-proven BKPyV-associated nephropathy; AUC: area under the curve; n: number of patients; *p: p*-values. Continuous data are expressed as medians and interquartile ranges. *(i) p*-values indicate the significance of the within-bp-BKPyVAN group difference before and after biopsy-proven BKPyVAN diagnosis in Fisher's exact tests or Wilcoxon matched-pairs signed-rank tests.

Supplementary data: Table-S2. Assessment of BKPyV and CEF-specific T-cell responses in the context of kidney transplantation (KTRs without BKPyV reactivation)

**Assessment of antiviral T-cell response**	Proliferation	IFN_{γ} secretion	TNF_{α} secretion	Co-secretion of TNF_{α} and IFN_{γ}	$7AAD^{+}$ target cells
BKPyV (LT-Ag and VP1) stimulation (n)	16	16	16	16	3
CD4 T cells (cell nb)	3.04 [1.22-3.83]	0.03 [0.03-2.03]	0.52 [0.03-1.87]	0.15 [0.03-0.75]	*NA*
CD8 T cells (cell nb)	3.76 [2.59-4.39]	0.03 [0.03-1.35]	0.66 [0.03-1.62]	0.032 [0.03-0.45]	5.48 [1.99-6.83]
Stimulation with other viral peptides (CEF peptide pool) (n)	10	13	13	13	-
CD8 T cells (cell nb)	3.90 [3.09-4.73]	2.16 [0.03-8.27]	0.48 [0.03-1.62]	3.49 [0.87-7.67]	-

BKPyV: BK polyomavirus; n: number of patients; CEF: pool of cytomegalovirus, Epstein Barr virus, and influenza virus peptides; cell nb: normalized cell number; NA: Not applicable. Continuous data are expressed as medians and interquartile ranges. For each patient, two BKPyV-specific responses were analyzed after stimulation with LT-Ag and VP1 peptides.

SUPPLEMENTARY MATERIAL – Supplementary methods

- KEY RESOURCES TABLE

REAGENT or RESOURCE	SOURCE	IDENTIFIER
• **Antibodies**		
BV605 Mouse Anti-Human CD3	BD Biosciences	CAT# 564712
VioGreen Mouse Anti-Human CD4	Miltenyi Biotec	CAT# 130-113-221
APC-Vio770 Mouse Anti-Human CD8	Miltenyi Biotec	CAT# 130-113-155
PE Mouse Anti-Human IFN-γ	Miltenyi Biotec	CAT# 130-113-493
Alexa Fluor 700 Mouse Anti-Human TNF-α	BD Biosciences	CAT# 557996
7-AAD	BD Biosciences	CAT# BD Biosciences
BV650 Mouse Anti-Human PD1	BD Biosciences	CAT# 564324
Biotin Mouse Anti-Human CTLA4	BD Biosciences	CAT# 555852
BV711 Streptavidin	BD Biosciences	CAT# 563262
APC Mouse Anti-Human TIM3	Miltenyi Biotec	CAT# 130-120-700
PE-Vio770 Mouse Anti-Human PD1	Miltenyi Biotec	CAT# 130-120-385
PE-Vio615 Mouse Anti-Human TIGIT	Miltenyi Biotec	CAT# 130-116-816
BV605 Mouse IgG2ak, Isotype Control	BD Biosciences	CAT# 562778
VioGreen Mouse IgG2ak, Isotype Control	Miltenyi Biotec	CAT# 130-113-841
APC-Vio770 Mouse IgG2ak, Isotype Control	Miltenyi Biotec	CAT# 130-113-832

PE Mouse IgG1, Isotype Control	Miltenyi Biotec	CAT# 130-113-762
APC Mouse IgG1, Isotype Control	Miltenyi Biotec	CAT# 130-113-758
Alexa Fluor 700 Mouse IgG1k, Isotype Control	BD Biosciences	CAT# 557882
BV650 Mouse IgG1k, Isotype Control	BD Biosciences	CAT# 563231
Biotin Mouse IgG2aκ, Isotype Control	BD Biosciences	CAT# 555572
PE-Vio770 recombinant human IgG1, REA Isotype Control	Miltenyi Biotec	CAT# 130-113-440
PE-Vio615 recombinant human IgG1, REA Isotype Control	Miltenyi Biotec	CAT# 130-113-439
• **Biological samples**		
Peripheral blood mononuclear cells	Isolated from patients included in this work	NA
• **Peptides and recombinant proteins**		
PepTivator BKV LT	Miltenyi Biotec	CAT# 130-131-249
PepTivator BKV VP1	Miltenyi Biotec	CAT# 130-131-251
PepTivator® CEF MHC Class I PLUS	Miltenyi Biotec	CAT# 130-098-426
Staphylococcal enterotoxin B (SEB)	Sigma-Aldrich	CAT# S4881
• **Commercial Reagents**		
BD Cytofix/Cytoperm™ Plus	BD Biosciences	CAT# 555028

Brefeldin A (BD GolgiPlug)	BD Biosciences	CAT# 555029
CellTrace™ CFSE	Thermo Fisher Scientific	CAT# C34554
Violet Proliferation Dye 450	BD Biosciences	CAT# 562158
CD4 MicroBeads, human	Miltenyi Biotec	CAT# 130-045-101
CD8 MicroBeads, human	Miltenyi Biotec	CAT# 130-045-201
BKV R-GENE	BioMerieux	https://www.biomerieux.fr/
HLA typing – One Lambda kits	Thermo Fisher Scientific	https://www.thermofisher.com/onelambda/wo/en/home.html
HLA typing – Olerup SSP kits	Bionobis	https://bionobis.com
• **Cytometer and Software**		
BD LSRFortessa™	BD Biosciences	https://www.bdbiosciences.com/
BD FACSDiva™ Software	BD Biosciences	https://www.bdbiosciences.com/
FlowJo (v10.7.1)	BD Biosciences	https://www.flowjo.com/
GraphPad Prism v9.2	GraphPad	https://www.graphpad.com/
Python v3.10.2	Python	https://www.python.org/

STATA v15.0	StataCorp	https://www.stata.com/company/

- SUBJECT DETAILS

Description of the cohorts

We performed a longitudinal observational study in the Kidney Transplant Department, University Hospitals, AP-HP. We assigned KTRs to four groups based on the level of BKPyV reactivation. The groups were defined as follows:

- Patients with BKPyV-DNAuria (urine BKPyV viral load > 200 copies/mL and plasma BKPyV viral load < 200 copies/mL in the last 12 months),
- Patients with BKPyV-DNAemia (stable or increasing plasma BKPyV viral load > 200 copies/mL in the last 6 months, without BKPyVAN diagnosis on kidney biopsy)
- Patients with bp-BKPyVAN(histologically proven BKPyVAN with a plasma BKPyV viral load > 200 copies/mL),
- And patients without BKPyV reactivation (plasma and urine BKPyV viral loads < 200 copies/mL in the last 12 months) as KTR group control.

We prospectively included 100 KTRs over the age of 18 years in the four groups (25 patients per group). The patients were followed prospectively between April 2014 and April 2018. Patients who had undergone combined multiple-organ transplantation were excluded, as were those with chronic viral infections, such as viral hepatitis or human immunodeficiency virus infection. KTRs with BKPyV reactivation that did not meet the study criteria were not included. The study was approved by the local Health Service Research Ethics Committee (Health Service Research Ethics Committee Ile de France VII ref. PP14-046) and performed following

the rules of the local ethics committee. Written informed consent was obtained from all patients. The research was conducted independently of sex or gender considerations.

We evaluated clinical characteristics, kidney graft outcome, immunosuppressive regimen, plasma and urine BKPyV viral load, HLA mismatches between donor and recipient, and BKPyV-specific T-cell functionality.

Collection of clinical and biological data

Demographic data for age and sex, the cause of end-stage renal disease, and dialysis and transplantation characteristics were recorded for all patients (Table 1).

We recorded median plasma and urine BKPyV viral loads and each patient's duration of BKPyV reactivation. Plasma and urine samples were obtained during routine visits to our center and during acute allograft failure. BKPyV screening was performed in accordance with international guidelines[1,2] by assessing plasma BKPyV load monthly in the first nine months after transplantation, and then every three months until two years after transplantation, and annually thereafter, with additional determinations in the event of an unexplained increase in serum creatinine concentration or after treatment for acute rejection. Urinary BKPyV load was also determined during plasma screening for BKPyV. We quantified BKPyV DNA by BKPyV-specific real-time PCR (BKV R-GENE).

Glomerular filtration rate was estimated with the MDRD-4 formula (eGFR) in the first month after transplantation, 12 months post-transplantation or at BKPyVAN diagnosis, and at the end of follow-up for each patient. The end of follow-up was defined as the occurrence of graft loss or when the last follow-up visit took place. We determined levels of tacrolimus, everolimus, and mycophenolate mofetil exposure (area under the curve – AUC in h.mg/L) before and after BKPyVAN diagnosis or 12 months after transplantation.

Histological assessment

Kidney biopsy was performed, in accordance with the transplantation protocol, three and 12 months after transplantation or in cases of acute kidney injury (increase in basal serum creatinine concentration of more than 25%). Bp-BKPyVAN was diagnosed histologically by the Histopathology Department in accordance with international guidelines. The lesions observed included enlarged nuclei with smudgy chromatin changes and intranuclear viral inclusions associated with a mononuclear inflammatory infiltrate and/or fibrosis. The presence of the virus was confirmed by immunohistochemistry with an antibody against large-SV40 tumor antigen.[12,13]

Assessment of HLA matching

We collected donor and recipient HLA typing data from the French organ allocation organization. For each donor-recipient combination, the degree of HLA matching was determined by counting the number of HLA-A, HLA-B, HLA-DR, and HLA-DQ antigenic mismatches between donor and recipient. We then assessed the effect of HLA matching on BKPyV reactivation and the occurrence of biopsy-proven BKPyVAN.

HLA typing was performed on recipients and living donors, with DNA molecular biology methods, using reverse sequence-specific oligonucleotides (SSOs) for the A/B/DRB1/DQB1 loci from the standard kit and from a high-definition kit from November 2016 onwards (One Lambda). HLA typing was performed for deceased donors with low-resolution sequence-specific primers (SSPs) for the A/B/DRB1/DQB1 loci (Olerup Reagents). These techniques provide at least split antigen-level resolution (most probable allele, depending on the method, locus, and antigen). If required, typings were extrapolated from low-resolution to high-resolution (two-field) using a machine learning tool trained on 60,000 patients typed with NGS in French laboratories.

Assessment of Grantham score

HLA evolutionary divergence between an individual's HLA alleles is a quantitative "pairwise" distance determined by the Grantham score,[14] in which the physiochemical properties of amino acids, and hence the functional similarity, are considered. It reflects the breadth of the immunopeptidome, defined as the set of peptides presented by MHC molecules on the surface of antigen-presenting cells to enable T-cell activation.

Antigenic mismatches within each locus are usually counted as 0, 1, or 2. However, not all HLA mismatches within a given locus are equally relevant. We, therefore, assessed donor-recipient HLA divergence by calculating the Grantham score,[14] which is usually used to quantify the divergence between two homologous alleles from an individual's single locus.[15–17]

The distance between two pairs of alleles was calculated as follows:

For a given gene, $i \in G = \{A, B, DRB1, DQB1\}$, $R_{i,1}, R_{i,2} \in \mathrm{A}_i$ denote the recipient alleles, and $D_{i,1}, D_{i,2} \in \mathrm{A}_i$ the donor alleles. A_i is the set of possible alleles for a given gene i. For each given gene $i \in G$, we can then define the function *Grantham Pair Score Sum*: $PSS_{i,Grantham} : \mathrm{A}_i^4 \rightarrow \mathrm{R}$

such that for $Ri,1, Ri,2, Di,1, Di,2 \in \mathrm{A}i$: $PSS_{i,Grantham} =$

$$min\left(Grantham(D_{i,1}, R_{i,1}), Grantham(D_{i,1}, R_{i,2})\right) +$$

$$min\ (Grantham(D_{i,2}, R_{i,1}), Grantham(D_{i,2}, R_{i,2}))$$

- EXPERIMENTAL MODEL AND METHOD DETAILS

Assessment of BKPyV-specific T-cell functionality

BKPyV-specific T-cell functionality was evaluated as reported previously (ref). All patients were assessed at inclusion.

We isolated peripheral blood mononuclear cells (PBMCs) from a whole-blood sample by centrifugation on a Ficoll gradient. PBMCs were frozen at a final concentration of 1×10^7 cells/mL and used within 12 months after cryopreservation.

For BKPyV activation, PBMCs were incubated with two different BKPyV-specific antigens (LT-Ag and VP1). These overlapping BKPyV-specific peptides activate both CD4 and CD8 T cells (PepTivator BKV LT-Ag or VP1 used at a final concentration of 1 µg/mL/peptide). We also used CEF peptides (pool of peptides from class I-restricted T-cell epitopes from cytomegalovirus, Epstein-Barr, and influenza viruses used at a final concentration of 1 µg/mL/peptide) as a global antiviral immune control. All activations were performed relative to a negative control (unstimulated cells) and a positive control (staphylococcal enterotoxin B – SEB, at a final concentration of 0.2 µg/mL). We assessed lymphocyte functionality by measuring proliferation, cytokine production, and cytotoxic capacities,[18] by flow cytometry (Figure S1). The lymphopenia of the KTRs resulted in low frequencies of BKPyV-specific T cells, as previously described,[11,50] and high rates of cell mortality during the freezing and thawing processes. We, therefore, assessed lymphocyte function for a limited number of KTRs in each group. Furthermore, we ensured that the analysis of BKPyV-specific T-cell responses was robust by analyzing a median of 40,492 [19,962-80,339] total T cells for CD4 T cells and 24,560 [11,443-65,902] total T cells for CD8 T cells, with a median of 590.5 [142.3-2,070] BKPyV-specific responding T cells. To confirm the specificity of the BKPyV-specific antigenic activation, we isolated BKPyV-specific CD8 T cells using BKPyV-specific pentamers from a subgroup of HLA-A2 or HLA-B7 KTRs. BKPyV-loaded pentamers were from ProImmune®. CD8 T cells were stained as recommended by the manufacturer.

T-cell proliferation in response to BKPyV antigens was assessed by CFSE dilution.

PBMCs (2x10^6/condition) were stained with 0.5 μM CFSE and incubated with BKPyV-specific peptide pools for five days. The stimulated cells were then stained with antibodies against surface markers (CD3-BV605, CD4-VioGreen, and CD8-APC-Vio770), and their proliferation capacity was evaluated by CFSE dilution (Figure S1).

T-cell cytokine production in response to BKPyV antigens

PBMCs (4x10^6/condition) were incubated overnight with BKPyV-specific peptide pools in the presence of brefeldin A (at a final concentration of 1 μL/mL). The stimulated cells were then stained with antibodies against surface markers (CD3-BV605, CD4-VioGreen, and CD8-APC-Vio770), fixed, permeabilized, and incubated with anti-interferon-γ (IFNγ) and anti-tumor necrosis factor-α (TNFα) antibodies (IFNγ-PE and TNFα-Alexa Fluor 700) (Figure S1).

T-cell cytotoxicity in response to BKPyV antigens

We measured BKPyV-specific CD8 T-cell cytotoxicity by incubating overnight CD8 T cells with autologous target cells (CD8-depleted PBMCs loaded with BKPyV peptides) (ratio 3/1). Target cells were distinguished from CD8 T cells by CFSE staining (0.5 μM). The mortality of target cells was evaluated by 7-amino-actinomycin D staining (0.5 μM) (Figure S1).

BKPyV-specific T-cell proliferation in the presence of allogeneic CD4 T-cell help

We assessed lymphocyte proliferation in response to BKPyV peptides in the presence and absence of allogeneic CD4 T cells. PBMCs from thirteen KTRs with bp-BKPyVANwere stained with a fluorescent dye, depleted of autologous CD4 T cells by magnetic depletion (CD4 MicroBeads), and incubated for five days with BKPyV-specific peptide pools in the presence of autologous or allogeneic third-party CD4 T cells, at a ratio of one CD4 to five non-CD4 T cells. We distinguished PBMCs from allogeneic third-party cells using two fluorescent dyes

(CFSE 0.5 µM or VPD450 0.5 µM). Unstimulated CD4-depleted PBMCs incubated in the presence of autologous CD4 T cells or allogeneic third-party CD4 T cells were used as controls. After five days of culture, the stimulated cells were stained for CD3, CD4, and CD8, and their proliferation capacity was evaluated by fluorescent dye dilution (CFSE or VPD450 - Figure 4b).

Assessment of T-cell inhibitory receptor expression

We evaluated the expression of lymphocyte inhibitory receptors (programmed cell death 1 - PD1, cytotoxic T-lymphocyte-associated protein 4 - CTLA4, T-cell immunoreceptor with Ig and ITIM domains - TIGIT; T-cell immunoglobulin and mucin domain-containing-3 - TIM3). In the first set of experiments, cytokine-secreting BKPyV-specific CD4 and CD8 T cells (Boolean gate from IFNγ- and/or TNFα-secreting cells) were assessed for the expression of PD1 and CTLA4 (PD1-BV650 and biotin CTLA4-BV711 Streptavidin), and compared to non-responding T cells (without cytokine production) (Figures S4). As the IFNγ production capacity was preserved in the bp-BKPyVAN group, we could perform a robust assessment of the BKPyV-specific T-cell phenotype. Thus, the median value was 162 [61;268] BKPyV-specific CD8 T cells for patients with BKPyV viremia and 111 [64;196] BKPyV-specific CD8 T cells for patients with BKPyVAN. In the second set of experiments, proliferative BKPyV-specific CD4 and CD8 T cells were assessed for the expression of PD1 and TIM3 and TIGIT (PD1-PeVio770, TIGIT-PeVio615, and TIM3-APC), and compared to non-responding T cells (non-proliferative T cells) (Figures S4).

- STATISTICAL ANALYSIS

Percentages and bar charts were used for categorical data. Medians and interquartile ranges were calculated for the analysis of continuous data. For the normalization of data distributions, the frequencies of BKPyV-specific T cells were subjected to natural logarithm or square root transformation. For categorical data, groups were compared in chi-squared tests. Kaplan-Meier

survival curves were plotted and compared in log-rank tests. For continuous data, comparisons between two groups were performed with nonparametric Mann-Whitney U or Wilcoxon matched-pairs signed rank tests as appropriate, whereas comparisons of more than two groups were conducted with nonparametric Kruskal-Wallis tests, followed by Dunn's test for multiple comparisons. All the tests used were two-sided. Correlations were evaluated with the nonparametric Spearman's rank correlation test. Odds ratios with 95% confidence intervals (OR [95% CI]) were estimated and compared by univariate multinomial logistic regression with correction for false discovery rate. Multivariate analysis was performed with Wald tests (multivariate multinomial logistic regression). Statistical analyses were performed with GraphPad Prism or STATA v15.0 (StataCorp, College Station, TX, US) software, and differences were considered significant if $p<0.05$.
